# Evolutionary Dynamics of Human Toll-Like Receptors and Their Different Contributions to Host Defense

**DOI:** 10.1371/journal.pgen.1000562

**Published:** 2009-07-17

**Authors:** Luis B. Barreiro, Meriem Ben-Ali, Hélène Quach, Guillaume Laval, Etienne Patin, Joseph K. Pickrell, Christiane Bouchier, Magali Tichit, Olivier Neyrolles, Brigitte Gicquel, Judith R. Kidd, Kenneth K. Kidd, Alexandre Alcaïs, Josiane Ragimbeau, Sandra Pellegrini, Laurent Abel, Jean-Laurent Casanova, Lluís Quintana-Murci

**Affiliations:** 1 Institut Pasteur, Human Evolutionary Genetics, CNRS, URA3012, Paris, France; 2 Department of Human Genetics, University of Chicago, Chicago, Illinois, United States of America; 3 Laboratory of Human Genetics of Infectious Diseases, Necker Branch, Institut National de la Santé et de la Recherche Médicale U550, Necker Medical School, Paris, France; 4 Institut Pasteur, Plate-forme Génomique, Pasteur Genopole, Paris, France; 5 IPBS/CNRS, Toulouse, France; 6 Institut Pasteur, Mycobacterial Genetics Unit, Paris, France; 7 Department of Genetics, Yale University School of Medicine, New Haven, Connecticut, United States of America; 8 University Paris René Descartes, Necker Medical School, Paris, France; 9 Institut Pasteur, Cytokine Signaling Unit, CNRS, URA1961, Paris, France; 10 Laboratory of Human Genetics of Infectious Diseases, Rockefeller Branch, The Rockefeller University, New York, New York, United States of America; University of Oxford, United Kingdom

## Abstract

Infectious diseases have been paramount among the threats to health and survival throughout human evolutionary history. Natural selection is therefore expected to act strongly on host defense genes, particularly on innate immunity genes whose products mediate the direct interaction between the host and the microbial environment. In insects and mammals, the Toll-like receptors (TLRs) appear to play a major role in initiating innate immune responses against microbes. In humans, however, it has been speculated that the set of TLRs could be redundant for protective immunity. We investigated how natural selection has acted upon human TLRs, as an approach to assess their level of biological redundancy. We sequenced the ten human TLRs in a panel of 158 individuals from various populations worldwide and found that the intracellular TLRs—activated by nucleic acids and particularly specialized in viral recognition—have evolved under strong purifying selection, indicating their essential non-redundant role in host survival. Conversely, the selective constraints on the TLRs expressed on the cell surface—activated by compounds other than nucleic acids—have been much more relaxed, with higher rates of damaging nonsynonymous and stop mutations tolerated, suggesting their higher redundancy. Finally, we tested whether TLRs have experienced spatially-varying selection in human populations and found that the region encompassing *TLR10*-*TLR1*-*TLR6* has been the target of recent positive selection among non-Africans. Our findings indicate that the different TLRs differ in their immunological redundancy, reflecting their distinct contributions to host defense. The insights gained in this study foster new hypotheses to be tested in clinical and epidemiological genetics of infectious disease.

## Author Summary

The detrimental effects of microbial infections have led to the evolution of a variety of host defense mechanisms. A vast array of host innate immunity receptors, critical sensors of viruses, bacteria, and fungi, exist to achieve permanent surveillance of intruding pathogens. The best characterized class of microbial sensors is the Toll-like receptor (TLR) family, which elicits inflammatory and antimicrobial responses after activation by microbial products. Here we investigated how microbes have exerted selective pressure on the human TLR family to gain insights on the extent to which they are functionally important in the immune system. By resequencing the ten TLRs in different worldwide populations, we show that intracellular TLRs—principally specialized in viral recognition—evolve under strong purifying selection, indicating their essential role in host survival, while the remaining TLRs display higher levels of immunological redundancy. However, for this latter group of genes, we also show that mutations altering immune responses have been in some cases beneficial for host survival, as attested by the signature of positive selection favoring a reduced TLR1-mediated response in Europeans. Our findings taken together indicate that the different human TLRs differ in their biological relevance and provide clues to be experimentally tested in clinical, immunological, and epidemiological studies.

## Introduction

Plants and animals have developed complex innate mechanisms to recognize and respond to attack by pathogenic microorganisms. The innate immune system is a universal and evolutionarily ancient mechanism at the front line of host defense against pathogens [1]–[3]. In vertebrates, invertebrate animals and plants, innate immunity relies on a diverse set of germline-encoded receptors referred to as pathogen- or pattern-recognition receptors (PRRs), or microbial sensors, which recognize molecular motifs shared by specific groups of microorganisms (often referred to as pathogen-associated molecular patterns or PAMPs) [2]–[6]. The last decade has seen a number of key advances in the understanding of PRR-mediated immunity, with Toll-like receptors (TLRs) being one of the largest and best studied families of PRRs [7]–[9]. The prototype of the TLR family is the *toll* gene in *Drosophila*, first identified for its role in dorso-ventral embryo patterning [10],[11] and later shown to be critical for effective immune responses in adult flies against fungi and Gram-positive bacteria [1],[12]. Since then, homologs of the Drosophila *toll* have been identified in many other species, with vertebrates typically having a repertoire of 10 to 12 TLRs [13],[14]. The role of mammalian TLRs in host defense has been studied mainly on the basis of their stimulation by different agonists *in vitro*, and knocked-out mice for one or several TLRs show variable susceptibility to various experimental infections [15]–[17]. Today, TLRs are known to respond to various pathogen-associated stimuli and transduce the signaling responses that are required for the activation of innate immunity effector mechanisms and the subsequent development of adaptive immunity [4],[9],[18].

In humans, the TLR family consists of 10 functional members (TLR1-TLR10) [4],[9],[14],[19],[20]. Human TLRs can be classified based on subcellular distribution: TLR3, TLR7, TLR8 and TLR9 are typically located in intracellular compartments such as the endosomes, whereas TLR1, TLR2, TLR4, TLR5 and TLR6 are generally expressed on the cell surface [4]. TLRs can be further classified based on known agonists. Intracellular TLRs sense nucleic acid-based agonists and are particularly specialized in viral recognition, whereas cell-surface expressed TLRs detect various other products, such as glycolipids, lipopeptides and flagellin, which are present in a large variety of organisms including bacteria, parasites and fungi [4],[21]. TLR10, which is expressed on the cell surface, remains the only orphan TLR member: its agonists and specific functions are currently unknown. The contribution of human TLRs to host defense in the course of natural, as opposed to experimental, infections has only recently begun to be deciphered. Patients with MyD88 or IRAK-4 deficiency, who do not respond to most TLR agonists with the exception of TLR3 and, to a lesser extent, TLR4 agonists, suffer from life-threatening infections caused by pyogenic bacteria [22]–[24]. Conversely, patients with UNC-93B deficiency, unresponsive to TLR3, TLR7, TLR8, and TLR9 stimulation, and patients with TLR3 deficiency, present with an apparently selective predisposition to herpes simplex virus-1 infection [25],[26]. Epidemiological genetics studies have investigated the contribution of genetic variation in TLRs, particularly for *TLR2*, *TLR4* and *TLR5*, to susceptibility to infectious diseases (for a review, see [27]). However, the clear involvement of these TLRs in the complex genetic control of infectious pathogenesis has not been unambiguously demonstrated in most cases.

The evolutionary genetics approach has increased our understanding of the evolutionary forces that affect the human genome providing an indispensable complement to clinical and epidemiological genetics approaches [28]–[30]. In the context of infection, identifying the extent and type of natural selection acting upon genes involved in immunity-related processes can provide insights into the mechanisms of host defense mediated by them as well as delineate those genes being essential in host defenses with respect to those exhibiting higher immunological redundancy [31]–[33]. In humans, the evolutionary approach has been successfully used for loci principally involved in adaptive immune responses or encoding for erythrocyte surface proteins (particularly malaria-related genes). For example, the excess of worldwide diversity at both HLA class I and II genes [34]–[37] and killer cell immunoglobulin-like receptors [38], as well as the high frequencies of the HbS allele [39]–[41] and the DARC null allele [42],[43] in Africa, clearly attest for the action of natural selection on host genes in response to pathogen presence. In the context of innate immunity, TLRs constitute the best characterized family of PRRs, yet the extent to which the different members of human TLR family have been subject to natural selection remains largely unknown. Furthermore, it has been speculated that the set of human TLRs could be redundant with other PRRs for protective immunity against most microbes [44]. Here, we investigated the evolutionary contribution of human TLRs to host defense by examining how natural selection has acted upon the different members of this family of microbial sensors in humans. We show that the different TLRs differ in their biological relevance and provide clues for their different contributions to host defense.

## Results

To assess whether and how natural selection has operated on human TLRs, we comprehensively re-sequenced the 10 TLR members in a panel of 158 healthy individuals originating from sub-Saharan Africa, from Europe and from East Asia. Each individual was sequenced for a total of 56.3 kb, 57.3% of which correspond to exonic regions, the rest comprising intronic and promoter regions (Table S1). In addition, to obtain an empirical framework of the expected levels of diversity at putatively neutrally-evolving loci, we further sequenced 20 independent noncoding genomic regions, each about 1.3 kb in size, dispersed throughout the genome, in the same multi-ethnic panel of individuals for whom we had sequenced the 10 TLR genes (Table S2, Materials and Methods for details). We first explored the evolutionary forces shaping TLR diversity since the divergence of the human and the chimpanzee lineages. Next, we tested for spatially varying selection among the different human populations, using several statistics summarizing the within and between-population data, including a newly-developed neutrality test that aims to identify alleles targeted by recent positive selection (i.e., ongoing-sweeps). Finally, we used predictive methods to generally assess the functional consequences of the nonsynonymous mutations (i.e., amino-acid replacement mutations) identified at the different TLRs, and performed *ad hoc* functional analyses to examine the effects of some TLR variants on the activation of the NF-κB signaling pathway.

### Global Genetic Diversity of the TLR Family in Human Populations

**Figure 1:**
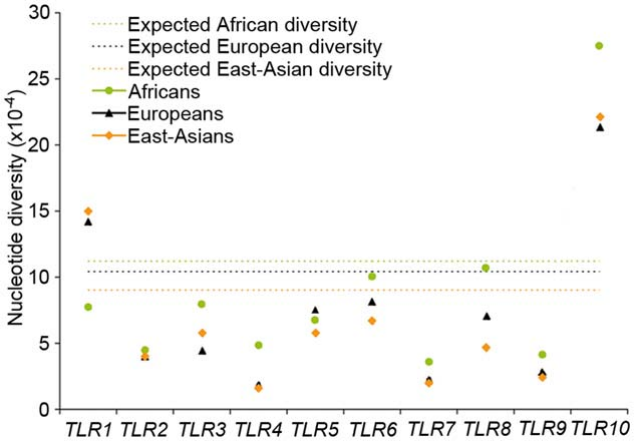
Global genetic diversity of the TLR family in human populations. Nucleotide diversity levels for the individual TLR genes in populations representing major ethnic groups. The expected diversity corresponds to the mean diversity levels observed for the 20 autosomal non-coding regions (“neutral” regions) in each geographical area.

Our population-based resequencing effort allowed us to identify 457 single nucleotide polymorphisms (SNPs), 103 of which corresponded to nonsynonymous mutations (Table S3). These extensive dataset provides with unbiased information on the number of tagging SNPs — the minimal set of SNPs needed to characterize haplotype diversity fully — required in each major population group in association studies concerning individual TLRs and infectious disease susceptibility (Table S4). In general, we observed large fluctuations in the overall levels of nucleotide diversity for the different TLR members (Figure 1, Table 1). Worldwide, *TLR4*, *TLR7* and *TLR9* were the least diverse, whereas *TLR10* was by far the most diverse gene with a 2-fold increase of general diversity with respect to the mean values observed for the twenty noncoding regions. For all TLR family members, with the exception of *TLR1*, genetic diversity was higher in Africans with respect to both Europeans and East Asians (Figure 1, Table 1), in agreement with the recent African origin of modern humans [45],[46]. At the haplotype level, we observed the same picture with Africans showing higher levels of haplotype diversity than non-African samples (Table 1). The only departure from this trend was observed at *TLR5*, which exhibited higher levels of haplotype diversity in non-Africans with respect to African populations.

**Table 1:** General diversity indexes for the 10 human TLR members sequenced in the panel of healthy individuals originating from different geographic areas worldwide.

	Africa (N = 126)^a^					Europe (N = 94)^a^					East-Asia (N = 96)^a^					Global (N = 316)^a^				
	S^b^	H^c^	NS^d^	π^e^	Hd^f^	S^b^	H^c^	NS^d^	π^e^	Hd^f^	S^b^	H^c^	NS^d^	π^e^	Hd^f^	S^b^	H^c^	NS^d^	π^e^	Hd^f^
***TLR1***	43	30	11	7.74	0.94	34	17	6	14.20	0.86	26	17	5	14.99	0.79	59	52	17	14.22	0.93
***TLR2***	16	16	2	4.48	0.84	12	11	5	4.01	0.81	6	8	0	4.00	0.76	24	23	7	4.70	0.86
***TLR3***	34	44	5	8.04	0.95	12	9	1	4.45	0.79	18	16	4	5.83	0.85	41	55	8	6.75	0.91
***TLR4***	50	41	10	4.92	0.91	12	12	3	1.83	0.66	15	16	2	1.60	0.70	66	59	13	3.12	0.80
***TLR5***	54	34	13	6.87	0.67	31	21	4	7.49	0.85	29	18	7	5.75	0.75	73	61	16	7.17	0.80
***TLR6***	34	27	12	10.06	0.91	15	10	2	8.12	0.79	16	11	7	6.69	0.73	44	36	17	9.63	0.89
***TLR7***	19	23	2	3.67	0.86	11	11	3	2.07	0.56	5	7	1	1.84	0.64	26	31	4	2.75	0.73
***TLR8***	25	29	1	10.70	0.95	16	19	0	7.11	0.83	16	11	0	4.61	0.50	33	46	1	9.63	0.87
***TLR9***	22	25	2	4.18	0.83	7	7	0	2.85	0.63	5	5	0	2.41	0.52	27	30	2	3.38	0.70
***TLR10***	59	38	13	27.49	0.96	44	17	13	21.50	0.74	39	12	8	22.08	0.75	64	55	18	26.26	0.90

a Number of chromosomes analyzed. For the X-linked *TLR7* and *TLR8*, the figures are 88, 68 and 74 for Africans, Europeans and East-Asians, respectively.

b Number of segregating sites (excluding indels).

c Number of haplotypes.

d Number of nonsynonymous mutations.

e Nucleotide diversity (×10^−4^).

f Haplotype diversity.

### Estimating the Direction and Strength of Selection in Human TLRs

To identify specific human TLR genes with evidence of selection since the divergence of the human and the chimpanzee lineages, we applied a statistical approach — the McDonald-Kreitman Poisson Random Field (MKPRF) test — that makes efficient use of McDonald-Kreitman (MK) contingency tables [47]–[49]. The MK contingency tables summarize the number of nonsynonymous and synonymous fixed differences between species (i.e., human and chimpanzee) and the number of nonsynonymous and synonymous polymorphisms within humans. Under neutrality, the ratio of the number of fixed differences between species to polymorphisms within species is expected to be the same for both nonsynonymous and synonymous mutations. Deviations from this expectation are indicative of natural selection: weak negative and/or balancing selection will result in an excess of nonsynonymous polymorphisms with regard to nonsynonymous divergence, and positive selection will lead to an excess of nonsynonymous divergence with respect to nonsynonymous polymorphisms. By explicitly taking into account shared parameters across genes (e.g., species divergence time), the MKPRF increases the power of the classical MK test and allows a more explicit estimation of the strength and direction of selection acting on individuals genes [48],[49]. The MKPRF enables the discovery of positive selection in evolutionarily constrained genes as well as the differentiation of weak from strong purifying selection. The parameters estimated by this method are ω, the ratio between the nonsynonymous and synonymous mutation rates, the species divergence time, and the selection coefficient (γ) of nonsynonymous polymorphisms (for details, see Material and Methods).

**Figure 2:**
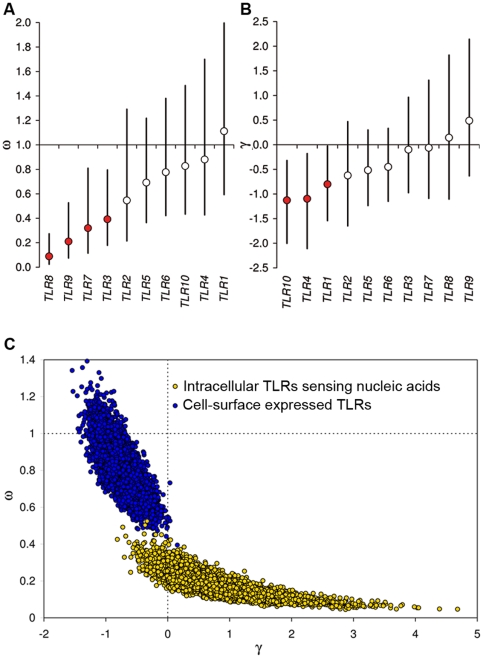
Estimation of the intensity of natural selection acting on individual TLR genes. (A) Strength of purifying selection acting on individual TLR genes, as measured by estimated ω values. Bars indicate 95% CIs and red circles indicate genes with ω estimates significantly lower than 1. (B) Strength of negative selection acting on individual TLR genes, as measured by the population selection coefficient γ. Bars indicate 95% CIs, and red circles indicate genes with γ estimates significantly lower than 0. (C) Joint estimates of the intensity of purifying (ω) and negative (γ) selection between intracellular nucleic acid sensors and cell-surface expressed TLRs.

We first estimated ω on individual TLR genes; ω (i.e., ω = log[θ_R_/θ_S_]) measures the selective constraint on amino-acid mutations. θ_R_ and θ_S_ are estimates of the rate of amino-acid replacement and silent mutations [47]–[50]. Under neutrality, ω is not significantly different from 1. Lower values are consistent with selection against nonsynonymous variants (purifying selection), whereas higher values reflect selection favoring amino-acid mutations (positive selection). With the exception of *TLR1* that presented an ω value of 1.11, all other TLRs had a posterior mean ω estimate lower than 1, suggesting that all these genes have been targeted by purifying selection to some extent. This type of selection eliminates almost all new nonsynonymous mutations from the population (θ_R_≪θ_S_) because their occurrence is not tolerated (e.g., lethal or strongly deleterious mutations) [28],[29]. Among the ten TLRs, *TLR3*, *TLR7*, *TLR8* and *TLR9* are those evolving under the strongest purifying selection, with ω values significantly lower than 1 (Figure 2A, Table S5). Interestingly, these four TLRs correspond to those receptors known to recognize nucleic acids and involved primarily in the recognition of viruses [21],[51]. This observation clearly demonstrates that these four TLRs have been subject to stronger purifying selection in humans with respect to the other TLR genes.

Next, we used the population selection parameter γ [47],[49] to identify TLR genes subject to selection operating on nonsynonymous mutations that are polymorphic in humans (i.e., non lethal mutations, Material and Methods). The parameter γ is negative if a gene displays excess of amino acid polymorphism within humans with respect to amino-acid divergence between species (weak negative and/or balancing selection). In contrast with purifying selection, weak negative selection does tolerate the occurrence of nonsynonymous mutations provided that they do not increase in frequency within the population (non lethal but slightly deleterious mutations) [28],[29]. Conversely, positive γ values reflect an excess of amino-acid divergence with respect to that observed for silent sites (positive selection) [49]. The posterior means of the γ draws for the different TLRs ranged from −1.13 to 0.49, with a clear tendency towards negative values (Figure 2B, Table S6). Nonetheless, only *TLR1*, *TLR4* and *TLR10* had the 95% confidence intervals entirely lower than neutral expectations (i.e., γ = 0). The excess of nonsynonymous polymorphism observed at these three genes could testify either an advantage to maintain functional diversity (balancing or frequency-dependent selection.) or the action of weak negative selection. However, most nonsynonymous variants are observed at very low population frequencies (nonsynonymous *vs.* silent mutations; χ^2^ test, P = 2.7×10^−3^, Figure S1), suggesting that weak negative selection is the most likely force operating on mutations causing amino-acid changes at these three genes. Taken together, our results revealed differences in the evolutionary constraint acting on TLRs: nucleic acid sensors (*TLR3*, *TLR7*, *TLR8* and *TLR9*), for which nonsynonymous mutations are most likely deleterious, and the remaining TLRs, which evolve to different extents under more relaxed selective constraints. This dichotomy was further supported by comparisons of joint estimates of the strength of purifying (ω) and negative (γ) selection between these two groups. Little overlap was found between the two distributions (Figure 2C). For TLRs sensing nucleic acids, the mean estimates of ω and γ were 0.17 [95% CI 0.09 to 0.31] and 0.83 [95% CI −0.11 to 2.2], respectively. For the cell-surface expressed TLRs, the mean estimates of ω and γ were 0.8 [95% CI 0.59 to 1.06] and −0.7 [95% CI −1.13 to −0.31], respectively. These results are therefore consistent with major differences in the intensity of selection acting on intracellular nucleic acid sensors with respect to the cell-surface expressed TLRs.

### Distribution of Fitness Effects of Nonsynonymous SNPs

**Figure 3:**
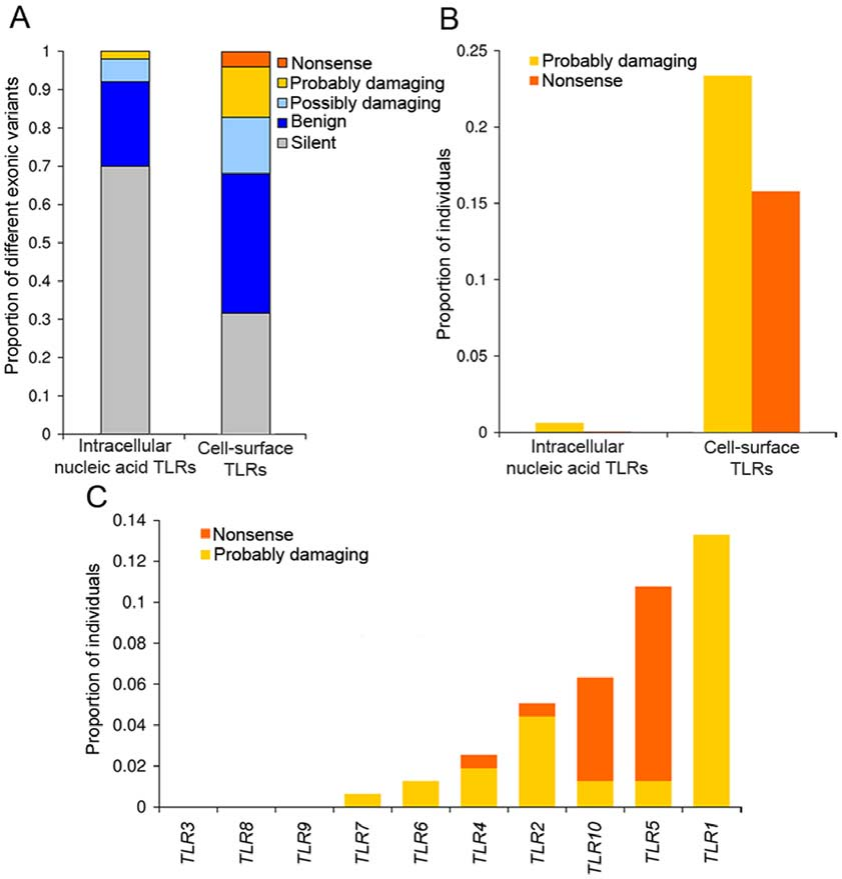
Functional diversity is unevenly distributed between the two groups of TLRs. (A) Distribution of the different classes of exonic polymorphisms (silent, stop, and the nonsynonymous variants considered to be benign, possibly damaging or probably damaging) between intracellular nucleic acid sensors and cell-surface expressed TLRs. (B) Proportion of individuals in the general population presenting a probably damaging or stop mutation in at least one TLR belonging from either of the two groups of TLRs. (C) Proportion of individuals in the general population presenting at least a probably damaging or a stop mutation for each individual TLR. The protein domains targeted by each of the nonsynonymous mutations identified are shown in Figure S9 for TLRs sensing nucleic acids, and in Figure S10 for the remaining TLRs.

The signature of strong purifying selection obtained for the intracellular TLRs sensing nucleic acids suggests that the corresponding genes can accumulate only synonymous mutations or mutations with no major effect on protein function in their exonic regions. Conversely, function-altering mutations are more likely to be present in the population for cell-surface expressed TLRs. We tested this hypothesis, by assessing the phenotypic consequences of the 103 nonsynonymous mutations identified in the 10 TLR genes, using the Polyphen algorithm [52] (Table S7). This method predicts the impact of nonsynonymous variants (benign, possibly damaging, or probably damaging) on the structure and function of the protein, using comparative and physical considerations including the analysis of multiple-species sequence alignments and protein 3D-structures [52]. We found that the different types of exonic mutations — synonymous, nonsynonymous variants considered benign, possibly damaging or probably damaging, and stop mutations — were unevenly distributed between the group of intracellular nucleic acids sensors and that of cell-surface TLRs (*χ^2^*-test, *P* = 1×10^−4^). Specifically, among the different exonic mutations identified in each group of TLRs, the proportion of possibly or probably damaging mutations and stop mutations observed on nucleic acids sensors was much lower (8%) than that observed for the remaining TLRs (32%) (Figure 3A). At the population level, we observed no probably damaging or stop mutations for nucleic acids sensors, with the exception of a single European individual presenting a probably damaging heterozygous *TLR7* mutation. Conversely, the proportion of individuals presenting probably damaging or stop mutations affecting at least one of the cell-surface TLRs was remarkably high (23% and 16%, respectively) (Figure 3B). A high proportion of individuals in the general population presented stop mutations affecting TLR2 (0.6%), TLR4 (0.6%), TLR5 (10%) or TLR10 (5%) (Figure 3C). The relatively high frequencies of probably damaging and stop mutations affecting cell-surface expressed TLRs most likely reflect their lesser essential role in protective immunity in the natural setting.

### Testing for Geographically Varying Selection among Human Populations

The previous inter-species analyses have proven to be powerful to identify classes of genes evolving under strong evolutionary constraints, however they are very much limited regarding the detection of more recent events of positive selection in human populations [29],[30],[53]. As a positively selected mutation increases in frequency in a given population (i.e., selective sweep), it leaves a distinct signature (e.g., skew in the distribution of allele frequencies) on the pattern of genomic variation in the immediate vicinity of the selected mutation [28],[29],[54]. To test for geographically varying selection among the different continental populations here studied, we first used several summary statistics of the within-population allele frequency distribution, including the commonly used Tajima's *D*, Fu and Li's F* and Fay and Wu's *H*. In total, we identified five TLRs in the African sample, three in the European sample and three in the East Asian sample, whose variation was not compatible with neutrality (Table 2). These observations could reflect selective pressures targeting different TLRs in different populations but could also result from the distinct demographic histories characterizing the different continental populations.

**Table 2:** Sequence-based neutrality tests for the 10 TLRs here studied.

Gene	Africa (N = 126)^a^						Europe (N = 94)^a^						East-Asia (N = 96)^a^					
	*TD* b	*P* c	*F* d	*P* c	*H* e	*P* c	*TD* b	*P* c	*F* d	*P* c	*H* e	*P* c	*TD* b	*P* c	*F* d	*P* c	*H* e	*P* c
***TLR1***	−1.47	NS	**−2.43** *	NS	−5.58	NS	0.45	NS	0.58	NS	1.4	NS	1.76	NS	1.38	NS	−0.6	NS
***TLR2***	−1.15	NS	**−2.56** *	NS	−1.07	NS	−0.94	NS	−1.65	NS	0.66	NS	0.68	NS	−0.35	NS	0.07	NS
***TLR3***	−0.76	NS	−0.52	NS	2.08	NS	0.29	NS	−0.65	NS	0.08	NS	−0.08	NS	0.51	NS	0.01	NS
***TLR4***	**−1.87** **	**0.021**	**−4.07** **	**0.017**	0.59	NS	−1.12	NS	−0.77	NS	−0.28	NS	**−1.65** *	**0.028**	**−3.18** **	**0.009**	−0.5	NS
***TLR5***	**−1.63** *	NS	−2.12	NS	**−11.58** **	0.0088	−0.49	NS	−0.43	NS	**−12** **	0.0164	−0.94	NS	−1.5	NS	**−10.77** **	0.0186
***TLR6***	−0.89	NS	−1.28	NS	−0,11	NS	0.61	NS	0.25	NS	−0.02	NS	−0.16	NS	−1.18	NS	−2.19	NS
***TLR7***	−1.34	NS	−0.99	NS	−0.24	NS	−1.38	NS	**−2.80** *	NS	0.93	NS	−0.01	NS	0.03	NS	0.81	NS
***TLR8***	0.92	NS	0.69	NS	−1.7	NS	0.84	NS	0.5	NS	−1.8	NS	−0.44	NS	−0.44	NS	−4.57	NS
***TLR9***	−1.02	NS	**−2.37** *	NS	−2.42	NS	0.72	NS	−0.13	NS	−1.72	NS	1.19	NS	−0.4	NS	−0.62	NS
***TLR10***	0.67	NS	0.19	NS	−5.25	NS	0.64	NS	0.95	NS	**−10.81** *	**0.053**	1.25	NS	**1.89** **	**0.012**	**−10.47** *	0.048

*: *P*<0.05.

**: *P*<0.02.

***: *P*<1×10^−3^.Values in bold refer to significant deviations from neutrality considering a constant size demographic model.

a Number of chromosomes analyzed. For the X-linked *TLR7* and *TLR8*, the figures are 88, 68 and 74 for Africans, Europeans and East-Asians, respectively.

b Tajima's *D*.

c *P* values calculated when considering Voight *et al.*'s [55] demographic model.

d Fu and Li's F*.

e Fay and Hu's *H*.

To account for demographic influences on the robustness of neutrality tests, we considered a demographic model previously validated using a set of 50 unlinked noncoding regions sequenced in a set of populations similar to ours (i.e., African, European and Asian) [55]. This model considers a bottleneck in non-African populations starting 40,000 years ago in an ancestral population of 9,450 individuals, and an exponential expansion in African populations (Material and Methods, for details on the demographic parameters used). This external demographic model fitted perfectly the patterns of neutral diversity (i.e., 20 noncoding regions) observed in our studied populations (Table S8). We thus reestimated the significance of all neutrality tests incorporating the demographic model of Voight *et al.* [55] into our neutral expectations. We found that *TLR4*, *TLR5*, *TLR7* and *TLR10* rejected neutrality for at least one statistical test (Table 2). Specifically, *TLR4* in Africans and East-Asians and *TLR7* in Europeans showed an excess of rare alleles, a pattern indicative of weak negative selection or a selective sweep. *TLR5* showed a significant excess of high-frequency derived variants in all population groups, compatible with the occurrence of a selective sweep. Similarly, *TLR10* showed a significant excess of high-frequency derived variants in East-Asians with the same trend observed for the European sample (*P* = 0.053).

**Figure 4:**
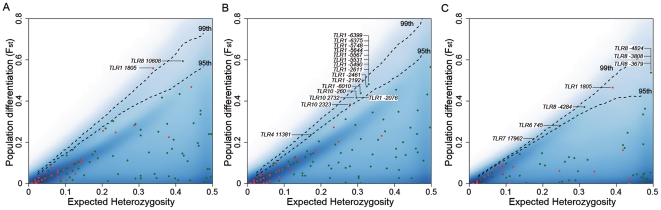
Some TLR members present extreme levels of population differentiation. (A) *F*_ST_ comparison for Africans *vs* Europeans (B) *F*_ST_ comparison for Africans *vs* East-Asians and (C) *F*_ST_ comparison for Europeans *vs* East-Asians. To account for possible differences in the allele frequency spectrum between our data and the HapMap genome-wide distribution [59], we compared *F*_ST_ as a function of heterozygosity. Green dots correspond to silent polymorphisms and red dots correspond to nonsynonymous mutations. The dashed lines represent the 95^th^ and 99^th^ percentiles of the HapMap genome-wide distribution (represented by the density area in blue). The significant values observed for *TLR7* and *TLR8* should be taken cautiously because these genes are located on the X chromosome and therefore, higher genetic drift may have exacerbated the levels of population differentiation observed.

An additional analytical approach to detect population-specific positive selection involves the comparison of genetic distances among populations, using the *F*_ST_ statistic [56],[57]. Specifically, local positive selection is known to increase the levels of population differentiation with respect to neutrally evolving loci [29],[30],[54],[55],[58]. We thus estimated the *F*_ST_ values for all SNPs identified in our study (457 SNPs) and compared them with the genomewide empirical distribution of *F*_ST_ obtained from the analysis ∼2.8 million HapMap Phase II SNPs [59]. Because the HapMap *F*_ST_ distribution includes loci targeted by positive selection [58], the comparison of TLRs *F*_ST_ against the HapMap distribution represents a highly conservative approach to detect selection (i.e., the “neutral” distribution also includes selected loci). In addition, we accounted for the differences in the allele frequency spectra between the sequence-based TLR dataset and the genotyping-based HapMap dataset, by comparing the *F*_ST_ values as a function of the expected heterozygosity (i.e., twice the product of allele frequencies). Our results revealed significantly high levels of population differentiation among several TLR variants, a large proportion of which corresponded to nonsynonymous mutations (Figure 4). Regardless of the populations' pairwise comparison, most TLR variants presenting high-*F*_ST_ were located in the cluster *TLR10*-*TLR1*-*TLR6* (66% of all high-*F*_ST_ SNPs) (Table S9).

### Evidence for the Action of Recent Positive Selection on the *TLR10*-*TLR1*-*TLR6* Cluster

**Figure 5:**
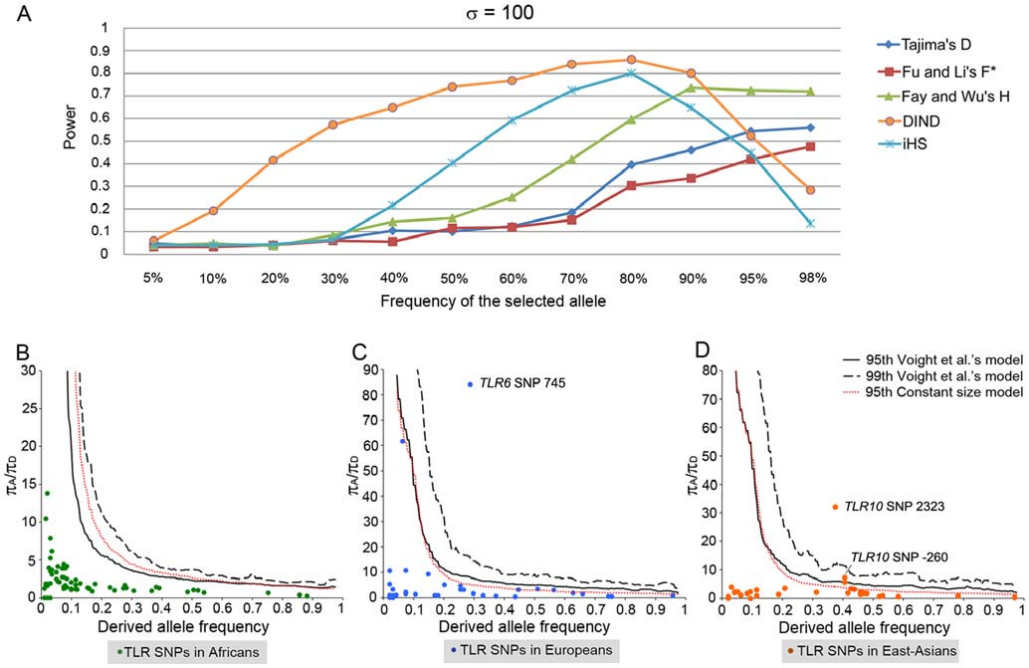
Signatures of positive selection targeting the *TLR10*-*TLR1*-*TLR6* gene cluster. (A) Power of the DIND test with respect to other statistics as a function of the frequency of the selected allele when the selection coefficient (S = 2Ns) is set to be 100. DIND test in (B) Africans, (C) Europeans and (D) East-Asians. *P* values were obtained by comparing the *iπ_A_*/*iπ_D_* values for the TLRs against the expected *iπ_A_*/*iπ_D_* values obtained from 10,000 simulations considering both a model of constant population size and the demographic model of Voight *et al.*'s [55] (Materials and Methods).

The *TLR10*-*TLR1*-*TLR6* gene cluster is located in a ∼60 kb genomic region in chromosome 4p14, the three genes being in strong linkage disequilibrium (LD) particularly in non-African populations (Figure S2). Because of the close vicinity of these genes, we performed a sliding-window analysis of nucleotide diversity π, Tajima's *D* and Fay and Wu's *H* across this region. These analyses revealed multiple windows in *TLR1* and *TLR10* showing significant deviations from neutral expectations, particularly among non-Africans (Figure S3). This observation, together with the high-*F*_ST_ values observed in this region, strongly suggests the occurrence of population specific events of positive selection. To identify more precisely the allele(s)/haplotype(s) being targeted by selection, we developed a new statistic — the Derived Intra-allelic Nucleotide Diversity (DIND) test — that makes maximum profit of resequencing data (see Materials and Methods for details). The rationale of this test is that, under neutral conditions, a derived allele that is at high population frequencies should present high levels of nucleotide diversity at linked sites (i.e., high levels of diversity within the class of haplotypes defined by the presence of the derived allele). Conversely, a derived allele that is positively selected will increase in frequency much quicker than the time needed to accumulate diversity at linked sites; a derived allele targeted by positive selection will be at high population frequencies but associated with low nucleotide diversity at linked sites. We first evaluated the power of the DIND test with respect to other commonly used frequency- and LD-based neutrality tests (i.e., Tajimas's *D*, Fu and Li's F*, Fay and Wu's *H* and iHS). Our simulations revealed that the DIND test clearly outperformed the other tests, particularly when the selected allele is found at a population frequency <70% (Figure 5A and Figure S4). The power of the test drops only when the selected allele is observed at near-fixation. Thus, the DIND test is especially useful for the identification of ongoing sweeps.

We applied the DIND test to our data by plotting for all SNPs identified in the *TLR10*-*TLR1*-*TLR6* region, the ratio between the ancestral and the derived internal nucleotide diversity (*iπ_A_*/*iπ_D_*) against the frequencies of the derived alleles (Figures 5B–5D). An elevated *iπ_A_*/*iπ_D_* associated with a high frequency of the derived allele is indicative of positive selection targeting the derived allelic state. Our analyses identified three mutations characterizing several *TLR10*-*TLR1*-*TLR6* haplotypes showing clear signs of positive selection: the nonsynonymous mutation C745T in TLR6 (P249S) tagging a single haplotype in Europeans (H34) (Figure 5C), and the nonsynonymous mutation A2323G (I775V) and the non-coding mutation G-260A, both in TLR10 (Figure 5D), defining three evolutionarily-related haplotypes in East-Asians (H41,54,55) (Figure S5). The action of positive selection targeting this gene cluster is further reinforced by the fact that, when using the HapMap data [59],[60], the haplotypes containing the selected alleles are also associated with significantly high levels of LD, as measured by the Long Range Haplotype (LRH) test [61] (Figure S6).

### Functional Analyses of the *TLR10-TLR1-TLR6* Gene Cluster

**Figure 6:**
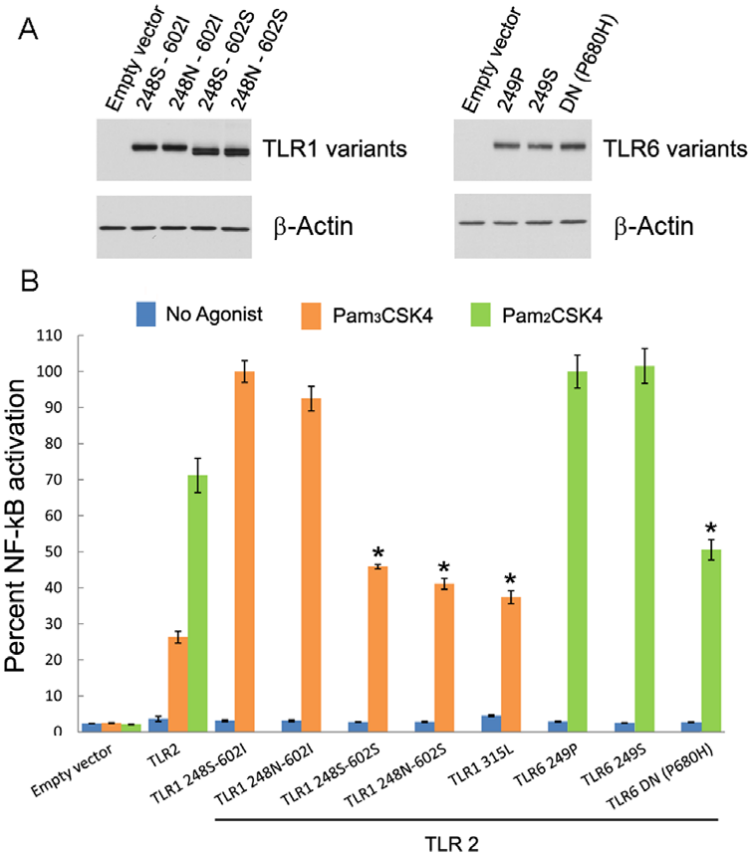
Functional analyses of the positively selected haplotype H34 in Europeans. (A) Level of expression of TLR1 and TLR6 variants. HEK 293T cells were transiently transfected with 100 ng of the indicated construct and whole cell lysates were analyzed by immunoblot with anti-HA tag antibody. (B) The TLR1 602S variant results in a diminished NF-kB signalling. HEK 293T cells were transiently co-transfected with an NF-κB reporter plasmid, a Renilla luciferase plasmid for transfection control and with TLR2 alone or in combination with the indicated TLR1 and TLR6 variants. One day after transfection, cells were stimulated with the indicated agonist at 10 ng/ml for 4 h and luciferase activities were measured. The data shown are the mean±SD of three replicates of a representative of three independent experiments, expressed as the percent relative firefly luciferase activity (RLU) (normalized to *Renilla* luciferase activity). * *p*<0.001 (as determined by Student's *t* test) when comparing the different TLR1 allelic variants with respect to the ancestral TLR1 248S-602I form, and when comparing the TLR6 variants with the ancestral TLR6 249P form.

The high frequencies of H34 in Europe (∼26%) and of H41-54-55 in Asia (∼40%) and the depicted signatures of positive selection (Figures 5 and Figure S6) suggest that these haplotypes harbor functional variation that has conferred a selective advantage among non-African populations. In Europe, H34 is characterized by three amino-acid changes: TLR1 S248N (SNP G743A), TLR1 I602S (T1805G) and TLR6 P249S (C745T).To assess the functional impact of each of these variants, we examined their respective effects on the activation of NF-κB signaling — the principal TLR-dependent pathway [62]. To do so, we generated by site-directed mutagenesis the three variants of H34 as well as the TLR1 P315L and the TLR6 P680H variants, which were shown to substantially diminish NF-κB activation [63]–[65]. All constructs were HA-tagged at the C-terminus. Since both TLR1 and TLR6 signal as heterodimers with TLR2, we co-transfected in human embryonic kidney (HEK) 293T cells the different *TLR1* and *TLR6* variants along with TLR2 and an NF-κB luciferase reporter plamid. The expression levels of the four TLR1 variants (248S/602I, 248N/602I, 248S/602S, 248N/602S) and the two TLR6 variants (249P, 249S) were found to be comparable (Figure 6A). Interestingly, variants containing the derived 602S allele migrated slightly faster most likely due to a polarity change (I602S). We next stimulated cells with their corresponding TLR agonists: PAM_3_CSK_4_, for the TLR1/2 heterodimer, or PAM_2_CSK_4_, for the TLR6/2 heterodimer (Figure 6B). In response to stimulation with PAM_3_CSK_4_, the ancestral TLR1 248S-602I form, when cotransfected with TLR2, mediated greater NF-κB activity than TLR2 alone (*P*<0.001). The ability of the 248N variant to mediate NF-κB signaling did not significantly differ from that of the ancestral form. By contrast, the derived 602S allele (1805G) presented impaired signaling with a drastic decrease of ∼60% of NF-κB activity in comparison with the 602I allele (Figure 6B), in agreement with previous studies [66],[67]. Consistently, the 248S and 248N in combination with either variant of 602 did not influence the degree of NF-κB activation. When cells were stimulated with the TLR6 PAM_2_CSK_4_ agonist, both the ancestral 249P and the derived 249S forms were similarly capable of mediating maximal NF-κB signaling (Figure 6B). Together, these results show that, among the three nonsynonymous variants of H34 (TLR1 248N, TLR1 602S and TLR6 249S), only TLR1 602S has a functional impact leading to impaired signaling.

In Asia, the putatively-selected haplotypes H41-54-55 are characterized by three aminoacid changes targeting TLR10 (N241H, I369L and I775V). We first observed that the three TLR10 variants were expressed at comparable levels (Figure S7). TLR10 is the only orphan TLR member for which no specific agonist has been yet identified. Several authors have evaluated the functional impact of some TLR mutations by over-expressing them and measuring NF-κB activity in the absence of stimulation [64],[68]. We found that neither the over-expression of TLR10 at different levels (see Material and Methods for details) nor the stimulation of transfected cells with TLR10 antibodies induce NF-κB activation for any of the variants tested nor for the wild-type haplotype (data not shown). As previously reported for TLR2 [68], our results showed that TLR10 do not induce NF-κB activation in the absence of stimulation, thus precluding us from evaluating the functional impact of TLR10 variants.

## Discussion

### The Evolutionary Dynamics of the Human TLR Family

The study of the evolutionary dynamics of the innate immune system is an excellent approach to test hypotheses concerning the evolution of genes mediating the antagonistic interaction between the host and the microbial environment. Here, we examined whether and how natural selection has targeted innate immunity receptors in humans, using as a paradigm the TLR family. Characterizing how rapidly, or not, innate immune genes evolve can increase our understanding of the recognition properties of these genes and the nature of the host-pathogen interactions mediated by them. In this respect, contrasting findings have been obtained for immunity genes in different model organisms [69]–[71, and references therein]. In the plant *Arabidopsis thaliana*, genes involved in the specific recognition of pathogen proteins (*R* genes) show little evidence of positive selection arguing against a coevolutionary arms race driving *R* gene evolution [72]. These genes display instead signatures of transient balancing selection causing high levels of protein variation maintained over intermediate periods of time [72],[73]. Conversely, no evidence for an important role of balancing selection has been found in *Drosophila* immunity proteins [69],[74],[75]. PRRs triggering humoral immunity (e.g., peptidoglycan recognition proteins) appear to evolve under purifying selection, while phagocytic receptors involved in cellular immunity (e.g., class C scavenger receptors) show evidence of ongoing positive selection [69], [75]–[77]. This observation suggests that, in *Drosophila*, the recognition properties of these two classes of immunity genes are quite distinct: PRRs recognize highly conserved microbial compounds and are therefore evolutionarily static, whereas phagocytic receptors may bind to evolutionarily labile pathogen molecules and are likely to coevolve with pathogens [69]. In humans, similarly to the data from *Drosophila* PRRs, we observed that TLRs, taken as a set, have evolved under the action of purifying selection. These results are consistent with a recent study that, based on a partially overlapping set of genes resequenced in an Indian population, proposes that purifying selection is the dominant signature among genes of innate immune system [78]. Conversely, our data do not support the notion that balancing selection is pervasive among human innate immunity genes, as it has been previously claimed [79]. Although strong evolutionary conservation is expected at PRRs that recognize conserved and essential molecular patterns of the microorganisms, our data revealed major differences in the intensity of selection acting upon the different members of the TLR family.

### Intracellular Nucleic Acids Sensors Are Essential and Non-Redundant in Host Survival

The biological relevance of the various TLR members can be inferred from the intensity of evolutionary constraints on these molecules. Our analyses clearly showed that the group of intracellular TLRs has been subject to strong purifying selection, whilst such a selective constraint appears to be less pronounced among cell-surface TLRs. This dichotomy most likely reflects both the different nature of the microorganisms targeted by the two groups of TLRs and the diverse spectra of targeted molecules displayed by the different microbes. Intracellular TLRs are principally involved in viral recognition through the sensing of their nucleic acids — the most dominant mechanism by which viruses are detected [21],[51]. Indeed, viral proteins serve as poor targets for innate recognition because they can rapidly evolve. To ensure effective viral detection, the host has bypassed this problem by using the intracellular TLR-mediated system, which targets various forms of viral nucleic acids (essential molecules that are difficult for the microorganism to alter). Conversely, cell-surface TLRs target multiple molecules (i.e., PAMPs) characterizing the structure or the metabolism of a plethora of microorganisms, mostly bacteria, parasites and fungi [4],[21]. Because these microbes display each several, different PAMPs (e.g., lipopolysaccharide, flagellin, etc), they can be simultaneously detected by different cell-surface TLRs, in contrast with viruses that are almost uniquely recognized by their nucleic acids. In this view, it is tempting to speculate that the extreme conservation observed at intracellular viral-recognition TLRs results from the very narrow choice these sensors have for targeting viral molecules (nucleic acids). More generally, this pattern of strong purifying selection suggests that viruses have globally exerted stronger selective pressure on these immunity sensors with respect to other microbes, consistent with the group of intracellular TLRs playing each a key role in host anti-viral defences. This hypothesis is supported by clinical genetics data indicating that TLR3 plays an essential role in natural immunity to herpes simplex virus-1 encephalitis [26]. With respect to TLR7, TLR8 and TLR9, although they have been proposed to be redundant against most common viruses [22]–[26],[80], individuals presenting TLR7, TLR8 or TLR9 deficiencies have never been reported, so a direct role of these genes in host anti-viral defenses cannot be ruled out. Overall, our data suggest that one or a few viruses (extinct and/or undiagnosed) have exerted pressure on TLR7, TLR8, and TLR9, but in a manner different from that of the ubiquitous herpes simplex virus-1, which exerts selective pressure on TLR3.

Viruses may not be the only selective pressure driving the selective maintenance of TLR3, TLR7, TLR8 and TLR9. Some TLRs appear to be involved in central nervous system development and maintenance [81],[82]. TLR8 has been implicated in neurite outgrowth in mouse, as neurons in mouse embryos have been shown to produce larger amounts of TLR8 during embryonic stages [82]. Interestingly, the human *TLR8* is the TLR under the strongest purifying selection (Figure 2A). Another factor that may have further contributed to the strong protein conservation of the four nucleic acid sensors is autoimmune avoidance. Indeed, the intracellular localization of TLR3, TLR7, TLR8 and TLR9 represents a mechanism for the host to prevent self nucleic acid recognition while preserving the ability to detect viral nucleic acids within the acidic environment of endosomes and lysosomes. Nevertheless, these TLRs can be stimulated “inappropriately” by certain endogenous RNA- and DNA-containing ligands [83],[84]. For example, plasmacytoid dendritic cells are activated, via TLR7 or TLR9, in response to “immune complexes” containing self DNA or RNA [83]. Furthermore, mice with an extra copy of *TLR7* have accelerated autoimmune reactions [85]. Conceivably, mutations increasing the reactivity of these TLRs to self nucleic acids or releasing them from the endosomal compartment would be highly detrimental, particularly during embryonic life, increasing selective constraints on these genes. Altogether, the strong purifying selection operating on *TLR3*, *TLR7*, *TLR8* and *TLR9* demonstrates their essential, non-redundant biological role in host survival.

### Cell-Surface TLRs Display Higher Evolutionary Flexibility

Unlike the TLRs sensing nucleic acids, the group of TLRs expressed at the cell surface — TLR1, TLR2, TLR4, TLR5, TLR6 and TLR10 — display higher evolutionary flexibility (i.e., lesser selective constraint on nonsynonymous mutations). The relatively high population-frequencies of nonsynonymous variants with probable effects on protein function or stop mutations on the corresponding genes (Figure 3C) suggest higher immunological redundancy. Similar patterns of segregating non-functional alleles have been reported in *Arabidopsis R* genes. Out of 27 *R* genes examined, 17 of them displayed high frequencies (up to 33%) of frameshift or stop codon mutations, reflecting complex episodes of balancing selection and relaxed constraint [72]. Likewise, the high prevalence of stop codons observed at some class-C scavenger receptors in *Drosophila* suggest higher redundancy, allowing increased frequencies of these non-functional alleles without compromising organismal fitness [77]. In humans, the higher redundancy we observed at cell-surface TLRs suggest that they have overlapping functions among them or with respect to other non-TLR sensors (e.g., C-type lectins). The most notable example of such redundancy is *TLR5*. The loss-of-function TLR5 392stop mutation is present at high population-frequencies (up to 23% in Europe and South Asia, see also [86]). Some stop mutations have been reported to confer a selectively advantage in humans [87],[88], with cases involved in immunity-related processes such as the truncated form of the caspase-12 gene [89]. However, no signal of recent positive selection was detected in the *TLR5* coding region (our data and [86]). This finding, as we previously reported for the innate immune receptor MBL2 [90], is consistent with a largely redundant role of TLR5 and suggests that other accessory mechanisms of pathogen recognition provide sufficient protection against infection. Our results support the notion that duality in sensing microbes, and therefore redundancy, may be a common feature among innate immunity receptors [91]. However, the higher biological dispensability of cell-surface TLRs does not exclude their important role in protective immunity. Indeed, our data revealed that weak negative selection precludes increases in the frequency of nonsynonymous variants in the population, at least for *TLR1*, *TLR4* and *TLR10* (Figures 2B and Figure S1). For *TLR4*, the action of weak negative selection is further reinforced by an excess of rare alleles as revealed by the significant negative values of Tajima's *D* and Fu & Li's F* (Table 2). Taken together, the nonsynonymous mutations accumulated by these genes, although non lethal, may have at least mildly deleterious phenotypic effects, as previously proposed for *TLR4* [92]. This prediction is further supported by the genetic association of some of these amino acid-altering mutations with susceptibility to a number of infectious diseases [27].

On the other hand, the higher evolutionary flexibility of the cell-surface TLRs can result in an increased number of potential targets for positive selection. In particular circumstances, mutations at these TLRs are not only tolerated but indeed positively selected either worldwide or in a population-specific manner. Consistent with a selective sweep at the worldwide level, *TLR5* displayed a significant excess of high-frequency derived variants (i.e., significantly negative Fay and Wu's *H*), restricted to its 5′-UTR (Figure S8). Although population structure can also result in significantly negative Fay and Wu's *H* values [93], this possibility is unlikely given that we observed a signal of selection in all studied populations. Given the present-day apparent redundant role of TLR5, as attested by high frequency of the TLR5 392stop mutation, we speculate that this selective sweep occurred in a more distant past; probably in a period characterized by a different set of pathogens against which certain *TLR5* variants were advantageous. TLR5 represents therefore a paradigm of the evolutionary dynamics that may characterize a large number of innate immune receptors; these genes may swing between being essential for protective immunity and becoming redundant in the natural setting depending on the temporally-varying microbial milieu. Finally, our analyses revealed that positive selection can also act locally at some cell-surface TLRs, leading to differential selection of resistance alleles in specific populations. Specifically, we identified two haplotypic backgrounds in the genomic region encompassing *TLR10-TLR1-TLR6* showing clear signs of positive selection in Europeans and East-Asians (Figures 5 and Figure S6).

### Has a Diminished TLR1-Mediated Response Conferred a Selective Advantage in Europeans?

Our data show that *TLR1*, and more specifically the nonsynonymous T1805G variant (I602S), is the genuine target of positive selection detected in the *TLR10-TLR1-TLR6* gene cluster in Europeans. First, *TLR1* is ∼2 times more diverse in non-African than in African populations, a pattern not compatible with the African origin of modern humans [45]. This pattern has been observed only once among the 323 genes (0.3%) sequenced by the Seattle SNP consortium. Thus, the increased diversity observed in *TLR1* among non-Africans probably results from ongoing hitchhiking between the selected allele and neutral variation at linked sites. Second, the 1805G (602S) mutation presents the highest level of population differentiation (*F*_ST_ = 0.54) of all SNPs located in this gene cluster (Figure 4, Table S9). Third, among the three nonsynonymous variants composing the haplotype identified as being under positive selection in Europeans (H34, see Figure S5), only the TLR1 1805G (602S) variant has a remarkable impairment effect on agonist-induced NF-κB activation, showing a decreased signaling by up to 60% (Figure 6B). These findings are consistent with previous studies showing that, homozygous, and to a lesser extent heterozygous, individuals for the 1805G allele present impaired TLR1-mediated immune responses after whole blood stimulation [66],[67],[94]. Taken together, it is tempting to speculate that an attenuated TLR1-mediated signaling, and a consequently reduced inflammatory response, has conferred a selective advantage in Europeans — a scenario that would explain the very high frequency (51%) of the “hypo-responsiveness” T1805G mutation in Europe. This observation raises questions about the possible evolutionary conflict between developing optimal mechanisms of pathogen recognition by TLRs, and more generally PRRs, and avoiding an excessive inflammatory response that can be harmful for the host.

### Conclusion

Our study has revealed that the mode and intensity of natural selection differs among the different TLR members, both at the species-wide level (all humans) and in a population-specific manner. Our results indicate that TLRs sensing nucleic acids play an essential, non-redundant role in host survival, either via protective immunity against viral infections (present or past), or because of their additional involvement in other non immunity-related processes of major biological relevance, or both. The strong selective constraints affecting these sensors suggest that mutations leading to impaired responses for these receptors are associated with severe clinical phenotypes. These genes are thus ideal candidates for involvement in individual Mendelian deficiencies (monogenic inheritance), as already shown for TLR3 deficiency [26]. Conversely, the relaxation of the selective constraints affecting cell-surface expressed TLRs, as illustrated by the higher rates of nonsynonymous and stop mutations, shows a higher level of biological redundancy for these receptors. Despite impaired responses involving these receptors have a more modest impact on human survival, polymorphism in these genes is involved in fine-tuning host defenses and may, therefore, subtly modulate individual susceptibility to infectious disease in the general population. Moreover, we show that impaired TLR-mediated responses may be in some cases beneficial for human survival, as attested by the signature of positive selection targeting the hypo-responsiveness *TLR1* 1805G allele in Europeans. Taken together, our evolutionary findings provide clues onto how variation in human TLRs may result in different contributions to the outcome of infectious diseases. More generally, the paradigm of TLRs neatly illustrates the value of integrating evolutionary genetic data into a clinical and epidemiological framework, for better definition of the ecological relevance of host defense genes to past and present survival *in natura*.

## Materials and Methods

### Population Samples

Sequence variation for the 10 members of the human TLR family and for the 20 autosomal non-coding regions was determined for a total of 158 individuals (316 chromosomes) representing major geographical regions. The descriptions of the specific population samples can be found in ALFRED (http://alfred.med.yale.edu) using the unique IDs given in parentheses. Sub-Saharan Africans were represented by Yorubans from Nigeria (31 individuals from UID “SA000036J”) and Chaggas from Tanzania (32 individuals from UID “SA000487T”); Europeans were represented by Danes (23 individuals from UID “SA000046K”) and Chuvash from Russia (24 individuals from UID “SA000491O”), and East-Asians were represented by Han Chinese (24 individuals from UID “SA000009J”) and Japanese (24 individuals from UID “SA000010B”). In addition, the orthologous regions of the TLR genes were sequenced in two chimpanzees, when the corresponding sequences were not publicly available. All individuals were healthy donors from whom informed consent was obtained. This study was approved by the Institut Pasteur Institutional Review Board (n° RBM 2008.06).

### Molecular Analyses

For each TLR, the totality of the exonic region and an at least an equivalent amount of non-exonic regions, including ∼1,000 bp of their promoter regions (i.e., upstream of the first transcribed exon), were sequenced (Table S1). Intronless genes like *TLR6* and *TLR9* were sequenced in their totality including ∼1,000 bp of their promoter regions. The 20 autosomal non-coding regions dispersed throughout the genome () and used as baseline of neutral diversity were chosen (i) to be independent from each other (ii) to be at least 200 kb apart from any known gene, predicted gene or spliced expressed sequenced tag (EST), and (iii) not to be in LD with any known gene or spliced EST. All sequences were obtained using the Big Dye terminator kit and the 3730 automated sequencer from Applied Biosystems. Sequence files and chromatograms were inspected using the GENALYS software [95]. As a measure of quality control, and to avoid allele-specific amplification, when new mutations were identified in primer binding regions, new primers were designed and sequence reactions were repeated. All singletons or ambiguous polymorphisms were systematically reamplified and resequenced.

### Estimation of General Diversity Indices

Haplotype reconstruction was performed by means of the Bayesian statistical method implemented in Phase (v.2.1.1) [96]. We applied the algorithm five times, using different randomly generated seeds, and consistent results were obtained across runs. Tagging SNPs for each population were selected using Haploview's Tagger in pairwise tagging mode (*r* ^2^≥0.80, minor allele frequency cut-off = 5%, and other settings at default value). To assess the ancestral allelic state for each SNP, we aligned the human sequence with genomes of other primates (*Pan troglodytes*, *Pongo pygmaeus*, *Macacca mullata*; UCSC database) and deduced by parsimony the ancestral state of each SNP. The different summary statistics such as the number of segregating sites (S), haplotype diversity (Hd), the average number of pairwise differences (π), and the sequence-based neutrality tests, such as Tajima's *D*, Fu and Li's F* and Fay and Wu's *H* tests were performed using DnaSP package v. 4.1 [97]. The sliding-windows of nucleotide diversity levels (π), Tajima's *D*, and Fay and Wu's *H* were also performed using DnaSP [97]. The size of each window was 1,500 nucleotides, and the step size was 500 nucleotides.

### Coalescent Simulations and Demographic Models

*P-values* for the various tests of neutrality were estimated from 10^4^ coalescent simulations under a finite-site neutral model and considering the recombination rate reported for the genes studied by deCODE map rates [98]. Coalescent simulations were performed using the program SIMCOAL 2.0 [99]. Each of the 10^4^ coalescent simulations was conditional on the observed sample size and the number of segregating sites observed in each gene and each of the sliding windows. To correct for the effects of demography on diversity patterns, we considered a validated demographic model that used also resequencing data of non-coding regions in set of populations similar to ours (i.e., African, European and Asian) [55]. Specifically, they estimated demographic parameters, based on 50 unlinked non-coding genomic regions resequenced in 45 individuals from three human populations (15 Hausa, 15 central Italians and 15 Han Chinese) [55]. We simulated non-African bottlenecks, conditionally on our European and East-Asian sample sizes (48 and 47 individuals, respectively), using their combination of parameters — i.e., a bottleneck starting 40,000 YBP in an ancestral population of 9,450 individuals with combinations of bottleneck duration and severity corresponding to the confidence region of parameter space with *P*-values of 0.05 (Figures 2A and 2D of ref. [55]). In addition, we also used their combination of parameters to simulate an African expansion, conditionally on our African sample size (63 individuals) — i.e., combinations of start of growth and grow rate of the exponential expansion corresponding to the confidence region of parameter space with *P*-values of 0.05 (Supporting Figure 3 of ref. [55]). To evaluate whether Voight *et al.*'s demographic model fitted our data, we simulated 1,000 sets of 20 regions each (the number of non-coding regions sequenced herein). Each simulated fragment had 1,300 bp, corresponding to the mean size of non-coding regions sequenced in this study (Table S2), with a per site mutation rate (*μ*) sampled from a Gamma distribution with a mean of 2.0×10^−8^ and with 95% of the draws varying from 1.5×10^−8^ to 4.0×10^−8^ [ref. 55]. We then tested whether the observed mean values for the different statistics observed for our non-coding regions was in the 95% confidence interval of the mean values estimated through this simulation procedure. We showed that Voight *et al.*'s demographic model fits well the patterns of diversity observed for the 20 non-coding regions sequenced in this study (Table S8). Once the model was validated, *P*-values of the summary statistics for the different TLRs were then corrected for this demographic model [55] by counting the number of simulated values of the different summary statistics that did not fall into the range of observed values (Table 2).

### Estimation of the Intensity of Purifying and Negative Selection

To model purifying/negative and directional positive selection operating on the different TLRs, we employed a Markov Chain Monte Carlo (MCMC) algorithm for the Bayesian analysis of polymorphism and divergence data under a Poisson random field setting [48],[49]. The different parameters are estimated by means of MK contingency tables comparing the levels of human polymorphism and human-chimpanzee divergence at silent and nonsynonymous sites [100]. This method assumes that a fraction, 1−*f_0_*, of the amino acid substitutions is lethal and never contributes to polymorphism or divergence. Consequently, the effective mutation rate at amino acid-altering sites after purifying selection is θ_r_/2 = 2*N_e_*μ*L_r_ f_0_*, where *L_r_* is the number of nucleotide sites at which a mutation would generate an amino acid change, *N_e_* the effective population size and μ the mutation rate per generation per site. Silent mutations are considered to be neutral, so that θ_s_/2 = 2*N_e_*μ*L_s_*, where *L_s_* is the number of nucleotide sites at which a mutation would not generate an amino acid change. Thus, given the data, we can estimate the locus scaled mutation rate for both nonsynonymous (θr) and synonymous sites (θs). The ratio θr/θs will be a direct proxy of the fraction, 1−*f_0_*, of the nonsynonymous mutations that have been eliminated from the population (i.e., the rate of purifying selection). In addition, and for each gene, the method quantifies the extent and directionality of selection operating on non-lethal nonsynonymous mutations in terms of the population genetic selection parameter (γ = 2*N*_e_ *s*) (specific details on the method can be found in refs. [47]–[49]). The model equally estimates the parameter τ, which corresponds to the number of generations since humans and chimpanzees started to diverge. Thus, each gene has its own γ, θr and θs values while τ is a shared parameter across loci. To approximate the posterior distribution of the parameters given the observed data (i.e., the joint probability for parameter values given the data), we used the MCMC method as already reported [48]. Specifically, for each TLR, we ran 10 independent MCMC chains with overdispersed starting points for 110,000 iterations. We retained samples after the 1,000^th^ step in each chain to allow for “burn-in” of the chain and used every 10^th^ sample from the chain as a quasi-independent draw. Thus, all posterior probabilities reported here are from 10,000 retained draws from 10 MCMC chains. To measure convergence, we used the Gelman Rubin statistics that was close to 1 for all parameters, illustrating that the 10 chains had converged before we retained our samples.

### Prediction of the Functional Impact of Nonsynonymous Mutations

The fitness status of all amino acid-altering mutations (i.e., benign, possibly damaging and probably damaging) was predicted using the Polyphen algorithm [52]. This method, which considers protein structure and/or sequence conservation information for each gene, has been shown to be the best predictor of the fitness effects of amino acid substitutions [101]. To independently assess the functional impact of these mutations, we replicated the analyses using the Panther algorithm [102]. Mutations predicted to be “probably damaging” (i.e., the most likely to affect protein function) by Polyphen were also predicted to be “strongly deleterious” using the Panther algorithm with a mean *P* deleterious = 0.90. The identification of the protein domains of the different TLR members was defined using the SMART program [103].

### Estimation of the Levels of Population Differentiation

Population differentiation was estimated by using the *F*_ST_ statistics derived from the analysis of variance (ANOVA) [56]. To identify SNPs presenting extreme levels of population differentiation, we compared the observed *F*_ST_ values at the level of individual SNPs in TLRs against the *F*_ST_ distribution [58] computed for ∼2.8 million HapMap Phase II SNPs [59]. Because *F*_ST_ values depend on allele frequencies, and the frequency spectrum of HapMap data is known to present a lack of low-frequency variants with respect to resequencing data [104], we compared *F*_ST_ values between TLRs and the HapMap data by comparing SNPs presenting similar allele frequencies (i.e., similar expected heterozygosity). Empirical *P* values for each SNP at the TLRs were estimated as follows: (i) we compared the *F*_ST_ values of each TLR SNP with those observed for HapMap SNPs presenting similar heterozygosity values (i.e., ±0.025 with respect to the observed value), (ii) among these frequency-matched SNPs, we estimated the proportion of HapMap SNPs presenting *F*_ST_ values higher than that observed for our data. The 95^th^ and 99^th^ percentiles for the HapMap genome-wide *F*_ST_ distribution were estimated by using heterozygosity sliding windows of size 0.05 with increasing steps of 0.01.

### Derived Intra-Allelic Nucleotide Diversity (DIND) Test

Because the commonly used sequence-based neutrality tests have low power to detect selection, particularly if the selective events are too recent, we developed a new statistics that takes maximum profit of extensive resequencing data. The rationale of this test is that, under neutral conditions, a derived allele that is present at high population frequency is by definition old and therefore, it should be associated with high levels of intra-allelic nucleotide diversity (i.e., high levels of diversity within the class of haplotypes defined by the presence of the derived allele). Conversely, under a scenario of positive selection, a derived allele will increase in frequency in the population much quicker than the time required to accumulate intra-allelic diversity. Thus, a positively selected allele will be at high frequency in the population but associated with low internal diversity at linked sites. To test for such scenarios, we proceed as follows. Let a given sample of *n* individuals be sequenced for a given locus, and let *x* be a given polymorphism identified at this locus, with an ancestral allele at frequency *n_A_* and a derived allele at frequency *n_D_*. Then, we calculate the intra-allelic nucleotide diversity *iπ* of both alleles at SNP *x* using the following formulas:
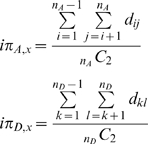
with *d_ij_* being the number of observed differences between the *i^th^* and *j^th^* haplotypes harboring the ancestral allele, *d_kl_* being the number of observed differences between the *k^th^* and *l^th^* haplotypes harboring the derived allele, and *n_A_C_2_* and *n_D_C_2_* the number of pairwise comparisons, respectively. The DIND test is based on the ratio *iπ_A_*/*iπ_D_* plotted against the frequency of the derived allele. A high *iπ_A_*/*iπ_D_* ratio (i.e., *iπ_D_*≪*iπ_A_*) together with a high frequency of the derived allele points to the action of positive selection (i.e., the internal diversity of the haplotypes associated with the derived allele is too small given the frequency of this allele in the population). For the particular situation in which iπD = 0, we attributed to the ratio *iπ_A_*/*iπ_D_* an arbitrary value corresponding to the maximal *iπ_A_*/*iπ_D_* value observed in the dataset plus 20. This decision was taken to avoid missing signals of selection resulting from situations where a highly frequent derived allele was associated with null intra-allelic diversity. To define statistical significance, the values estimated for TLRs were then compared against the background neutral distribution obtained by means of 10,000 simulations of the *TLR10*-*TLR1*-*TLR6* region, conditional on the number of segregating sites and the recombination rate of the region, and integrating the demographic model previously described.

### Power Calculations for the DIND Test

We used the program SelSim to simulate data with selection and recombination [105]. We simulated genomic regions of 60 kb and with 182 polymorphic sites — equivalent to the *TLR10-TLR1-TLR6* gene cluster — and a per locus recombination rate (4*Nr*) ρ = 1×10^−3^. The model used assumes that a new positively selected mutation experiences a constant additive selection pressure σ = 2Ns, where *N* is the population size and *s* is the additive selective advantage per copy per generation. The data are sampled when the mutation reaches a specified frequency. For each combination of σ and frequency of the selected site, we performed 250 simulations of 100 chromosomes each. Critical values for each statistic at *P* = 0.05 were obtained using identical simulations but with σ = 0.

### Long Range Haplotype (LRH) Test

To perform the LRH test [61], we retrieved from the HapMap data [59],[60] the haplotypes corresponding to the genomic region encompassing the *TLR10*-*TLR1*-*TLR6* gene cluster (1 Mb regions centered on TLR1). Then, we selected core regions identified as haplotype blocks (restricted to SNPs genotyped on these three genes), following the criteria of Gabriel *et al.* (2002) [106], and we assessed, for each core haplotype, its relative extended haplotype homozygozity (REHH) 200 kb apart. The only exception was for the European sample, where we defined core haplotypes as clusters of four continuous SNPs. This is because the SNP745 in *TLR6* (identified as being positively selected by the DIND test) was not included in any of the core haplotypes obtained using the criteria of Gabriel *et al.* (2002) [106] Thus, for this particular situation, we assessed, for each core haplotype, its REHH 300 kb apart to improve the power of the test. To test the significance of potentially selected core haplotypes, we compared the values obtained for our core regions with the empirical distribution of “core haplotype frequencies versus REHH” obtained from the screening of ∼50,000 core haplotypes from chromosome 4 in Yoruban, Asian and European-descent populations from HapMap data [59],[60]

### Plasmid Constructs and Site-Directed Mutagenesis

NF-κB luciferase reporter construct containing the luciferase gene under the control of six thymidine Kinase NF-κB sites from the thymidine kinase gene was kindly provided by Oreste Acuto. The *Renilla* Luciferase construct was purchased from Promega (Promega, Madison, WI). All TLRs constructs were purchased from Invivogen (InvivoGen, San Diego, CA). Allelic variants of *TLR1*, *TLR6*, and *TLR10* were made using the QuickChange Site Direct Mutagenesis system according to the manufacturer's instructions (Stratagene, La Jolla, CA). All constructs were systematically verified by sequencing of the complete TLR gene with a 3130×l Genetic Analyzer (Applied Biosystems, Foster City, CA).

### Cell Culture, Transfections, and Dual Luciferase Reporter Assays

The HEK 293T cell line was cultured in DMEM supplemented with 10% FBS, 100 IU penicillin, 100 µg/ml streptomycin (Invitrogen, Carlsbad, CA), at 37°C in a humidified incubator at 5% CO_2_. HEK 293T cells were seeded in 24-well plates (5×10^4^−1×10^5^ cells/well). The day after, cells (reaching 30–50% of confluency) were transiently transfected with a NF-κB reporter construct pNF-κB -luc from Stratagene, along with constructs expressing the various TLRs using FuGene 6 reagent from Roche Diagnostics according to the manufacture's recommendations. All plasmids used in transfections were purified using the Endofree plasmid kit (Qiagen, Chatworth, CA). Briefly, 100 ng of NF-κB reporter construct was cotransfected with TLR constructs and with 5 ng pRL-TK-*Renilla* luciferase construct (Promega, Madison, WI) as a control for transfection efficiency. For each transfection point, total DNA was adjusted to 300 ng with the use of the empty vector pcDNA3.1. For TLR2/1 and TLR2/6 transfection studies, 5 ng of TLR2 was cotransfected with 100 ng of TLR1 or TLR6. For TLR10 experiments, different concentrations of TLR10 variants were tested for the constitutive activation of NF-κB in the absence of stimulation (from 25 ng to 300 ng TLR10/well). After 24 h of transfection, cells were stimulated for 4 h with 10 ng/ml of Pam_3_CSK_4_ for TLR2/1 or Pam_2_CSK_4_ for TLR2/6 (EMC microcollections) in triplicate. No stimulation was performed for TLR10 because the ligand remains unknown. Then, supernatants were discarded and cells were lysed in 100 µl of passive lysis buffer (Promega, Madison, WI) and assayed for dual luciferase activities (Firefly and Renilla luciferase activities) according to the manufacturer's instructions. Luciferase activity was normalized by Renilla luciferase activity to account for the transfection efficiency and expressed as the mean relative stimulation±SD of three replicates of a representative of three independent experiments (each performed in triplicate).

### Protein Analysis

Cell lysates were subjected to SDS-PAGE (10%) under reducing conditions. Membranes were probed with an anti-HA tag antibody (Invivogen, San Diego, CA) followed by HRP-conjugated rabbit antimouse IgG (JacKson ImmunoResearch, West Grove, PA). Detection was performed with the Pierce Western blotting reagent (Thermo Scientific, Rockford, IL).

## Supporting Information

Figure S1 Allele frequency spectrum of silent and nonsynonymous mutations for the genes displaying signatures of weak negative selection (*TLR1*, *TLR4* and *TLR10*). A χ^2^-test was used to compare the proportion of SNPs with Minimum Allele Frequency (MAF)<0.05 between silent and nonsynonymous mutations at *TLR1*, *TLR4* and *TLR10*.
(0.12 MB DOC)

Figure S2 Linkage disequilibrium (LD) maps, based on D' values, for the *TLR10-TLR1-TLR6* genomic region. LD map in (A) African, (B) European, and (C) East-Asian populations. LD was estimated for SNPs with MAF>0.2. LD blocks were defined using the criteria of [106].
(2.15 MB DOC)

Figure S3 Multiple deviations from neutrality in the *TLR10*-*TLR1*-*TLR6* region. The scheme at the top provides a simplified view of the *TLR10*-*TLR1*-*TLR6* genomic region sequenced in this study. Genes are represented in a 3′-to-5′ orientation (minus strand). The thin line represents the intronic and promoter regions, small boxes refer to non-coding exons and large boxes refer to protein coding regions. Intergenic and non-coding sequence stretches that were not sequenced in this study are represented by their size in kilobases (i.e., 6.8 kb, 12.1 kb, 1.5 kb, 2.6 kb and 20.5 kb). (A) Sliding-window analysis of nucleotide diversity (π) across the *TLR10*-*TLR1*-*TLR6* region. The dashed lines denote the mean π values observed for the 20 non-coding regions in Africans (green), Europeans (black) and East-Asians (blue). (B) Sliding-window analysis of Tajima's *D* across the *TLR10*-*TLR1*-*TLR6* region in Africans (green), Europeans (black) and East-Asians (blue). (C) Sliding-window analysis of Fay and Hu's *H* across the *TLR10*-*TLR1*-*TLR6* region in Africans (green), Europeans (black) and East-Asians (blue). (B,C) Filled circles represent those windows significantly deviating from neutral expectations when considering the Voight *et al.*'s demographic model [55] (Materials and Methods). Single-SNP *F*_ST_ values for the population pairwise comparisons in (D) Africans vs Europeans, (E) Africans vs East-Asians and (F) Europeans vs East-Asians. The dashed lines correspond to the 95^th^ and 99^th^ percentiles of the *F*_ST_ values obtained from the 20 non-coding regions sequenced in the same individuals. Red dots correspond to nonsynonymous mutations.
(0.58 MB DOC)

Figure S4 Statistical power to detect ongoing sweeps at a *P*-value of 0.05, using various statistics. (A) Power of the various statistics when the selected allele is set to be at 30% frequency (similar to the frequency of the selected haplotypes identified at the *TLR10-TLR1-TLR6* cluster) and considering increasing selection coefficients. (B) Power of the various statistics when the selected allele is set to be at 80% frequency and considering increasing selection coefficients.
(0.37 MB DOC)

Figure S5 Inferred haplotypes for the *TLR10-TLR1-TLR6* gene cluster. Haplotype composition and frequency distribution in (A) Africans, (B) Europeans, and (C) East-Asians. The chimpanzee sequence was used to deduce the ancestral state at each position. Yellow columns correspond to nonsynonymous mutations. The frequency of each haplotype in the different populations studied is presented in the right of the figure. Haplotypes identified as being under positive selection by the DIND test are presented in red. Only haplotypes appearing more than once in each of the populations are shown.
(0.06 MB DOC)

Figure S6 Long Range Haplotype (LRH) test for the *TLR10-TLR1-TLR6* gene cluster. LRH in (A) Africans, (B) Europeans and (C) East-Asians. The haplotypes identified as being positively selected by this test correspond to the H26–31 in Africans, the H34 Europeans and the H41 and H55 in East-Asians, as presented in Figure S5. The same haplotypes in Europeans and East-Asians were identified as being under positive selection by using the DIND test (Figure 5).
(0.18 MB DOC)

Figure S7 Expression level of TLR10 variants. HEK 293T cells were transfected with (A) 25 ng, (B) 50 ng, (C) 100 ng and (D) 300 ng of the different *TLR10* variants. Equal volumes of each lysate were loaded on a 10% denaturing polyacrylamide gel. Membrane was probed with anti-HA tag antibody followed by HRP-conjugated rabbit antimouse IgG.
(0.14 MB DOC)

Figure S8 Sliding-window analysis of Fay and Wu's *H* across the *TLR5* genomic region. The size of each window was 1,000 nucleotides with a step size of 250 nucleotides. *P-values* were estimated from 10^4^ coalescent simulations under a finite-site neutral model conditional on the number of segregating sites observed in each of the sliding-windows. Filled circles represent those windows significantly deviating from neutral expectations when considering the validated demographic model (Materials and Methods).
(0.12 MB DOC)

Figure S9 Protein domain architecture of the intracellular TLRs sensing nucleic acids. Nonsynonymous mutations in black, blue and orange correspond to those considered as benign, possibly damaging and probably damaging. Variants in red correspond to stop mutations. The identification of the protein domains of the different TLR members was defined using the SMART program [103].
(7.00 MB DOC)

Figure S10 Protein domain architecture of the cell-surface expressed TLRs. Nonsynonymous mutations in black, blue and orange correspond to those considered as benign, possibly damaging and probably damaging. Variants in red correspond to stop mutations. The identification of the protein domains of the different TLR members was defined using the SMART program [103].
(8.20 MB DOC)

Table S1 Details on sequenced regions and fragments for the 10 human TLRs.
(0.28 MB DOC)

Table S2 Genomic features and mean diversity indices of the 20 independent noncoding genomic regions.
(0.10 MB DOC)

Table S3 Population allele frequencies for all SNPs identified among the 10 TLRs.
(0.15 MB XLS)

Table S4 Inferred haplotypes for each TLR and its corresponding tagging SNPs to be used in the different population groups to characterize SNPs with MAF>5%.
(0.48 MB XLS)

Table S5 Convergence and summary statistics of the marginal posterior distribution of ω estimations across 10 MCMC Chains with overdispersed starting points.
(0.04 MB DOC)

Table S6 Convergence and summary statistics of the marginal posterior distribution of γ estimations across 10 MCMC Chains with overdispersed starting points.
(0.04 MB DOC)

Table S7 Prediction of the fitness effect for nonsynonymous variants identified among the different TLRs.
(0.14 MB DOC)

Table S8 Expected mean values of sequenced-based neutrality tests considering the demographic model of Voight *et al.* (2005).
(0.04 MB DOC)

Table S9 List of high-*F*_ST_ SNPs observed in the 10 human TLRs.
(0.08 MB DOC)
