# Supplementary figures and images for: Evolutionary Dynamics of Human Toll-Like Receptors and Their Different Contributions to Host Defense
Source: PLoS Genet. 2009 Jul 17;5(7):e1000562. doi: 10.1371/journal.pgen.1000562 (PMC2702086; doi:10.1371/journal.pgen.1000562)

**Figure S1**


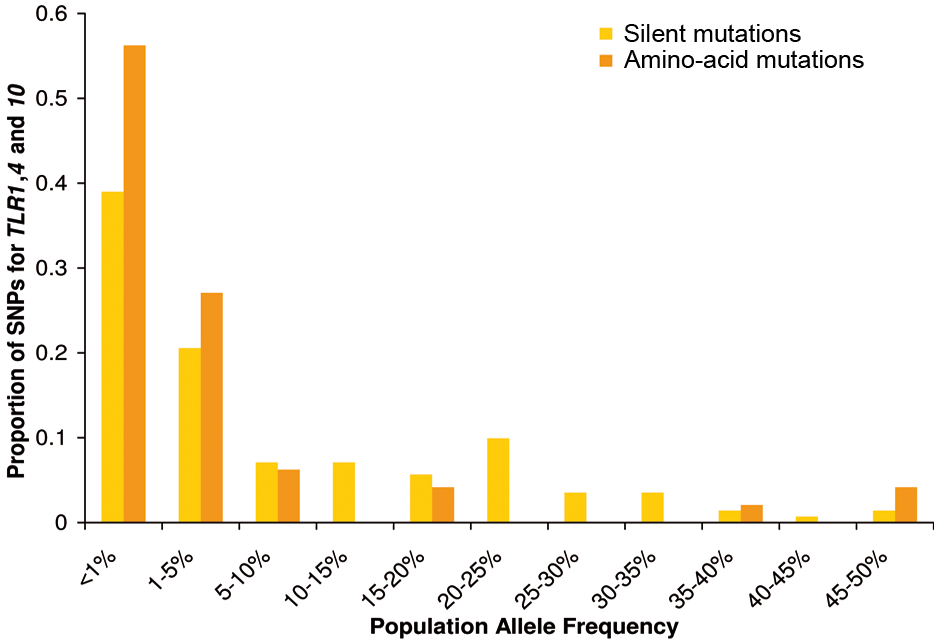

Supplement: Figure S1 — Allele frequency spectrum of silent and nonsynonymous mutations for the genes displaying signatures of weak negative selection (TLR1, TLR4 and TLR10). A χ2-test was used to compare the proportion of SNPs with Minimum Allele Frequency (MAF)<0.05 between silent and nonsynonymous mutations at TLR1, TLR4 and TLR10. (0.12 MB DOC) [file pgen.1000562.s001.doc]

**Figure S2**


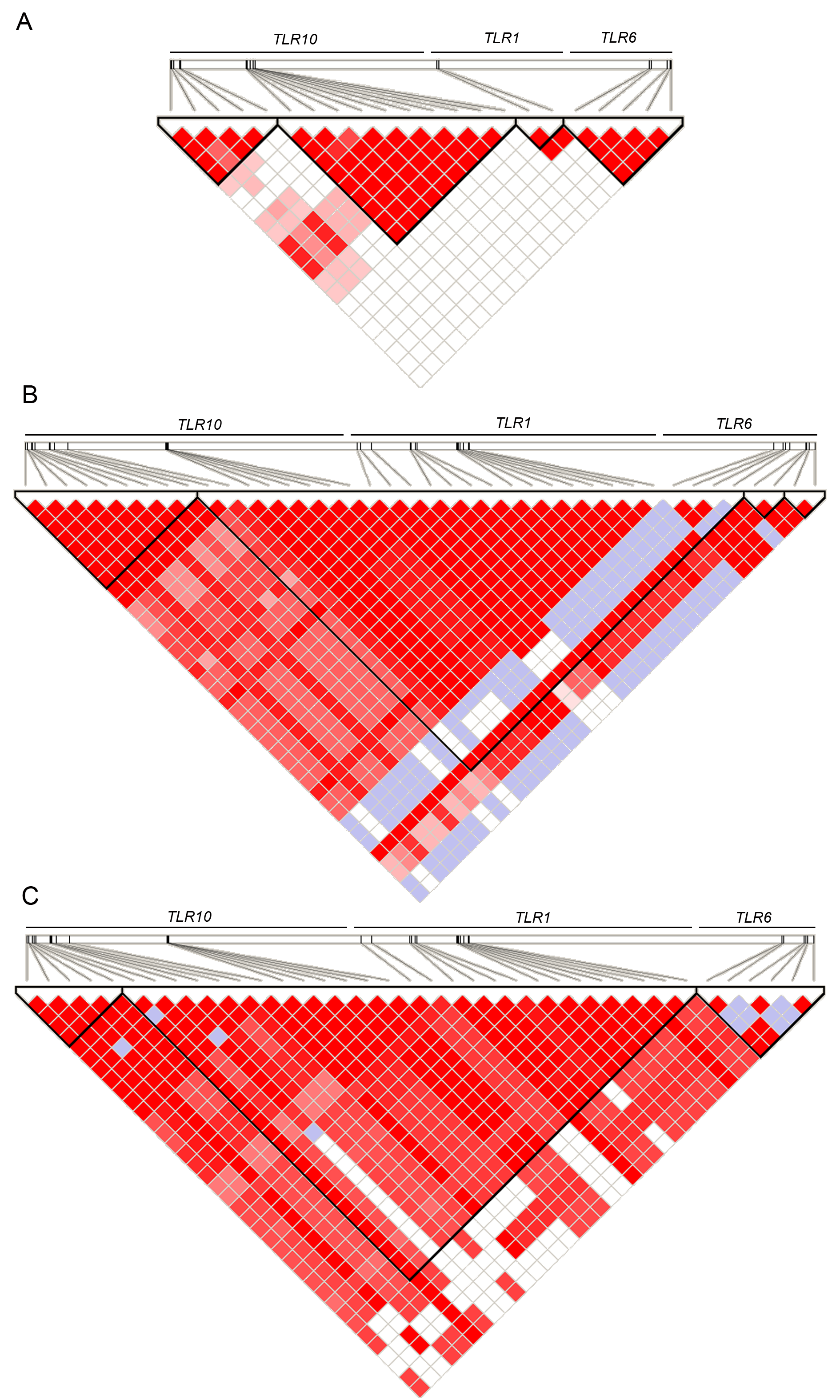

Supplement: Figure S2 — Linkage disequilibrium (LD) maps, based on D' values, for the TLR10-TLR1-TLR6 genomic region. LD map in (A) African, (B) European, and (C) East-Asian populations. LD was estimated for SNPs with MAF>0.2. LD blocks were defined using the criteria of [106]. (2.15 MB DOC) [file pgen.1000562.s002.doc]

**Figure S3**


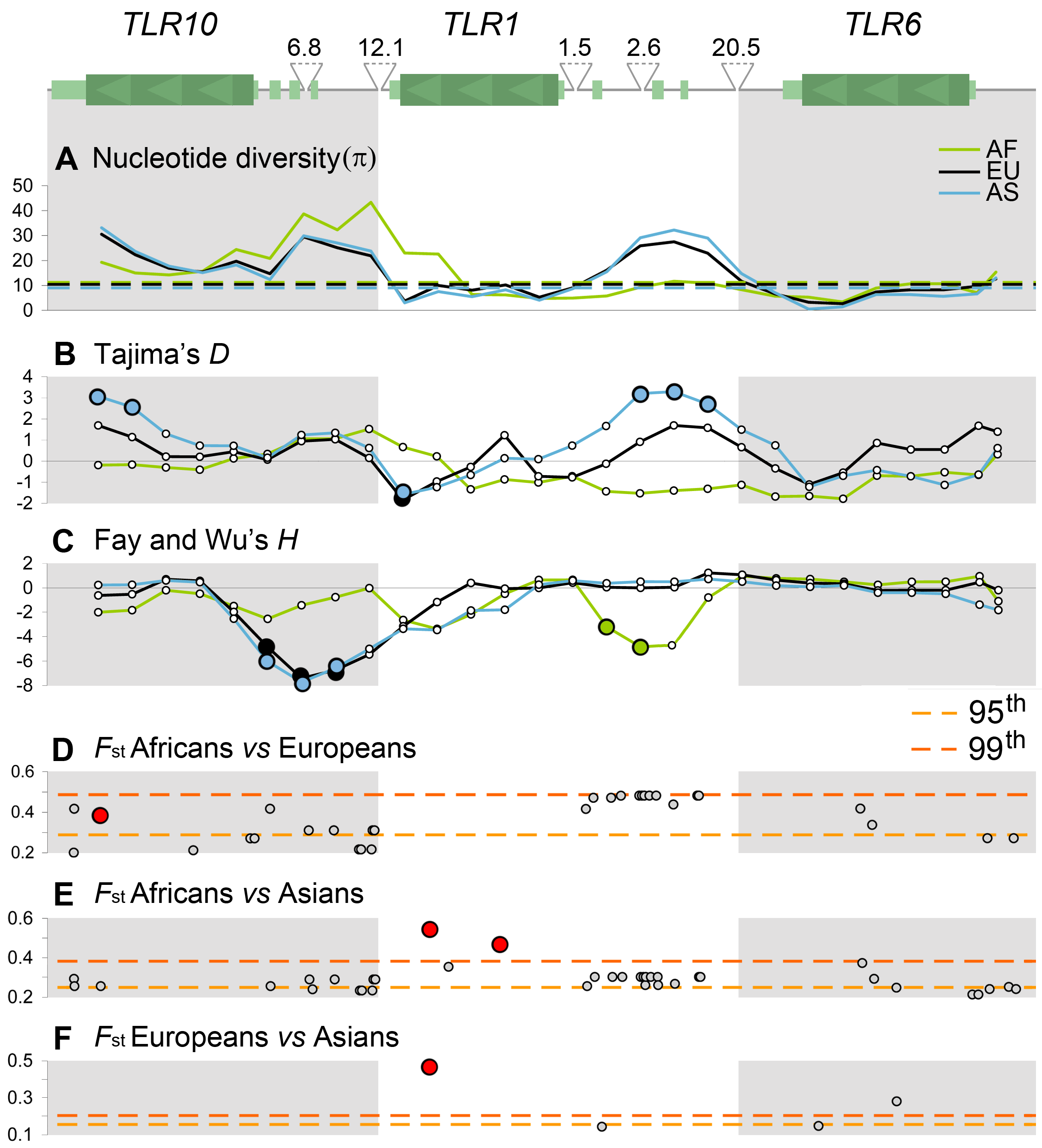

Supplement: Figure S3 — Multiple deviations from neutrality in the TLR10-TLR1-TLR6 region. The scheme at the top provides a simplified view of the TLR10-TLR1-TLR6 genomic region sequenced in this study. Genes are represented in a 3′-to-5′ orientation (minus strand). The thin line represents the intronic and promoter regions, small boxes refer to non-coding exons and large boxes refer to protein coding regions. Intergenic and non-coding sequence stretches that were not sequenced in this study are represented by their size in kilobases (i.e., 6.8 kb, 12.1 kb, 1.5 kb, 2.6 kb and 20.5 kb). (A) Sliding-window analysis of nucleotide diversity (π) across the TLR10-TLR1-TLR6 region. The dashed lines denote the mean π values observed for the 20 non-coding regions in Africans (green), Europeans (black) and East-Asians (blue). (B) Sliding-window analysis of Tajima's D across the TLR10-TLR1-TLR6 region in Africans (green), Europeans (black) and East-Asians (blue). (C) Sliding-window analysis of Fay and Hu's H across the TLR10-TLR1-TLR6 region in Africans (green), Europeans (black) and East-Asians (blue). (B,C) Filled circles represent those windows significantly deviating from neutral expectations when considering the Voight et al.'s demographic model [55] (Materials and Methods). Single-SNP F ST values for the population pairwise comparisons in (D) Africans vs Europeans, (E) Africans vs East-Asians and (F) Europeans vs East-Asians. The dashed lines correspond to the 95th and 99th percentiles of the F ST values obtained from the 20 non-coding regions sequenced in the same individuals. Red dots correspond to nonsynonymous mutations. (0.58 MB DOC) [file pgen.1000562.s003.doc]

**Figure S4**


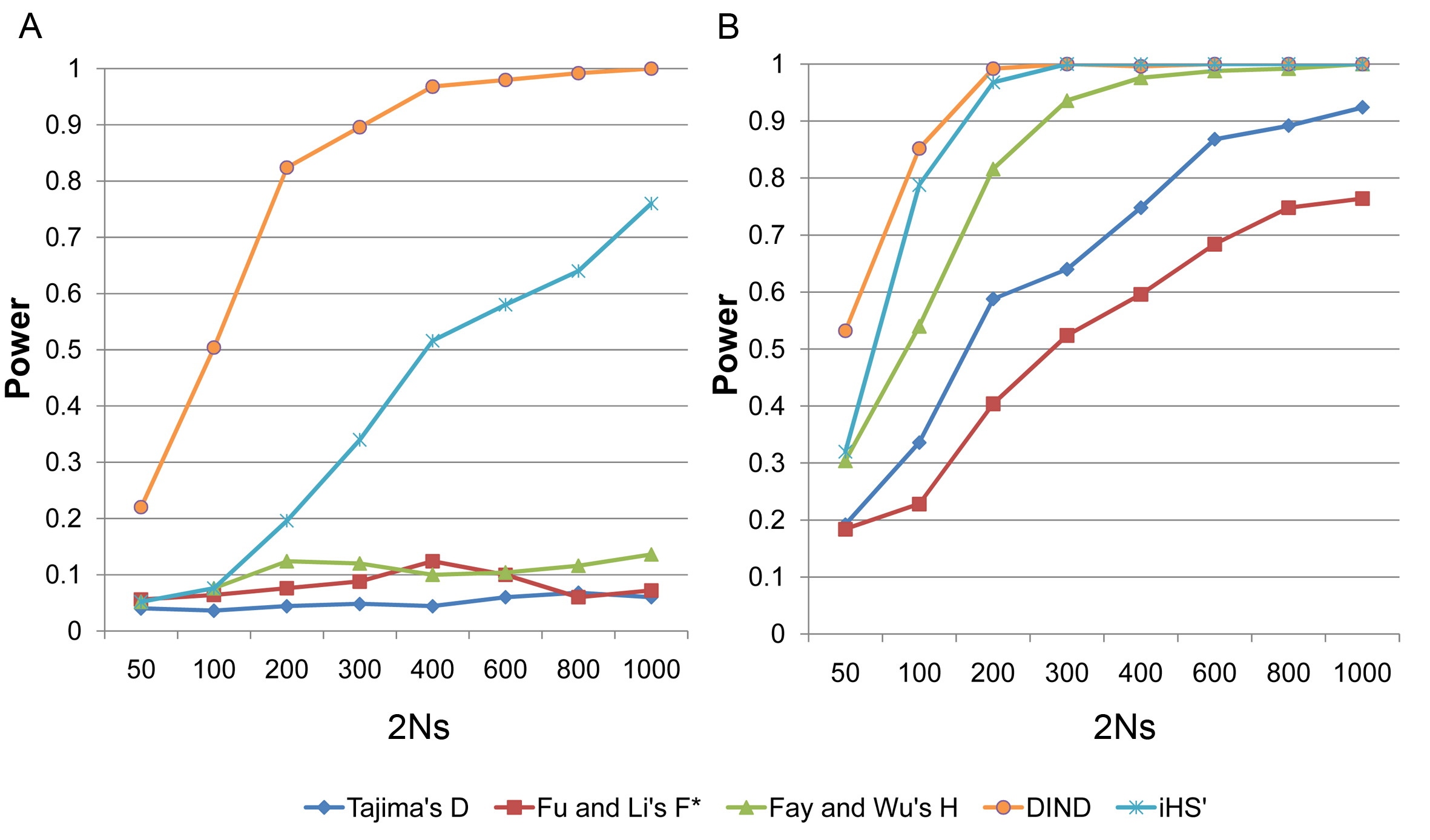

Supplement: Figure S4 — Statistical power to detect ongoing sweeps at a P-value of 0.05, using various statistics. (A) Power of the various statistics when the selected allele is set to be at 30% frequency (similar to the frequency of the selected haplotypes identified at the TLR10-TLR1-TLR6 cluster) and considering increasing selection coefficients. (B) Power of the various statistics when the selected allele is set to be at 80% frequency and considering increasing selection coefficients. (0.37 MB DOC) [file pgen.1000562.s004.doc]

**Figure S6**


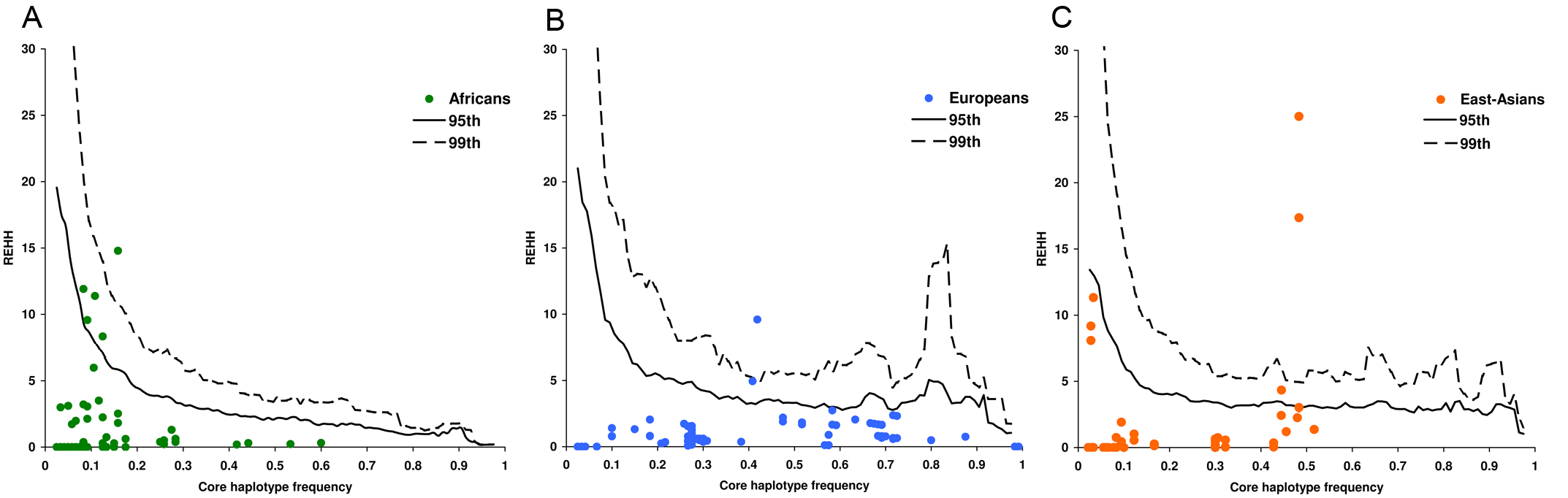

Supplement: Figure S6 — Long Range Haplotype (LRH) test for the TLR10-TLR1-TLR6 gene cluster. LRH in (A) Africans, (B) Europeans and (C) East-Asians. The haplotypes identified as being positively selected by this test correspond to the H26–31 in Africans, the H34 Europeans and the H41 and H55 in East-Asians, as presented in Figure S5. The same haplotypes in Europeans and East-Asians were identified as being under positive selection by using the DIND test (Figure 5). (0.18 MB DOC) [file pgen.1000562.s006.doc]

**Figure S7**

**
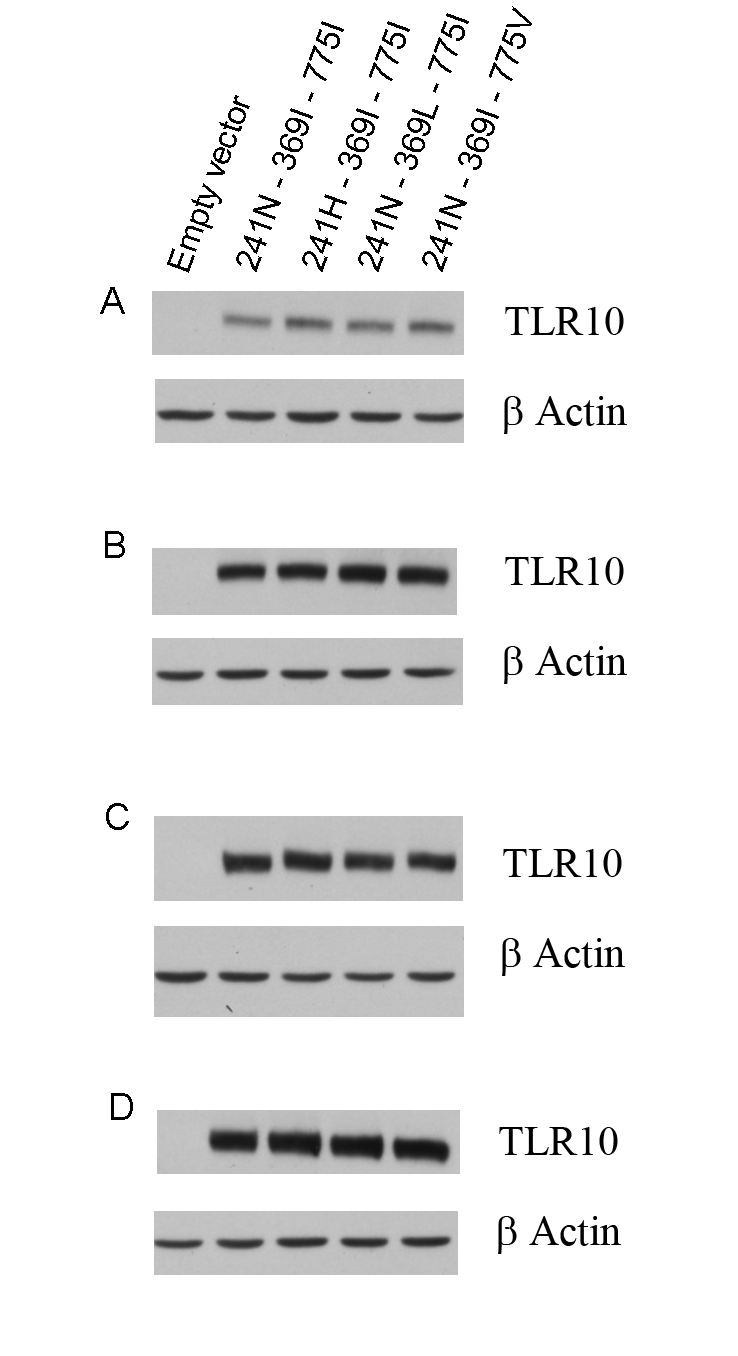
**

Supplement: Figure S7 — Expression level of TLR10 variants. HEK 293T cells were transfected with (A) 25 ng, (B) 50 ng, (C) 100 ng and (D) 300 ng of the different TLR10 variants. Equal volumes of each lysate were loaded on a 10% denaturing polyacrylamide gel. Membrane was probed with anti-HA tag antibody followed by HRP-conjugated rabbit antimouse IgG. (0.14 MB DOC) [file pgen.1000562.s007.doc]

**Figure S8**


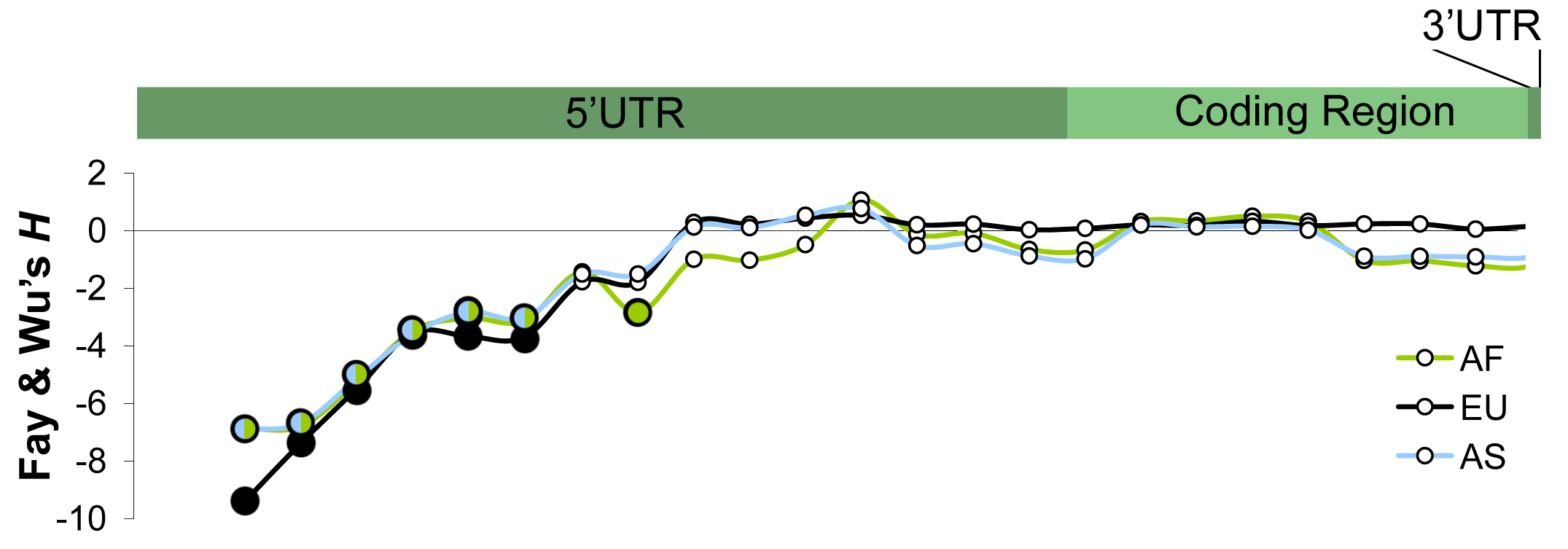

Supplement: Figure S8 — Sliding-window analysis of Fay and Wu's H across the TLR5 genomic region. The size of each window was 1,000 nucleotides with a step size of 250 nucleotides. P-values were estimated from 104 coalescent simulations under a finite-site neutral model conditional on the number of segregating sites observed in each of the sliding-windows. Filled circles represent those windows significantly deviating from neutral expectations when considering the validated demographic model (Materials and Methods). (0.12 MB DOC) [file pgen.1000562.s008.doc]

**Figure S9**


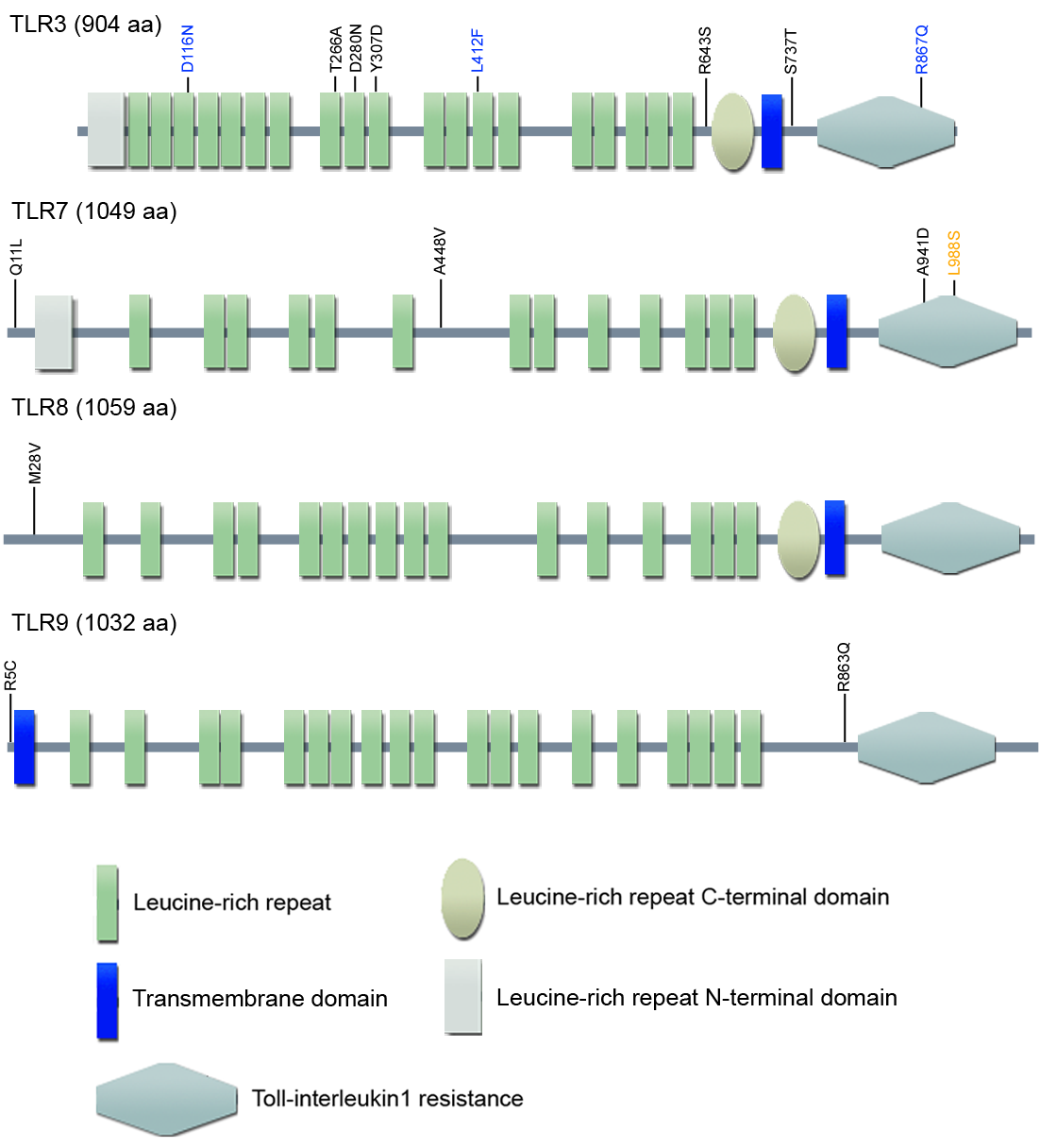

Supplement: Figure S9 — Protein domain architecture of the intracellular TLRs sensing nucleic acids. Nonsynonymous mutations in black, blue and orange correspond to those considered as benign, possibly damaging and probably damaging. Variants in red correspond to stop mutations. The identification of the protein domains of the different TLR members was defined using the SMART program [103]. (7.00 MB DOC) [file pgen.1000562.s009.doc]

**Figure S10**


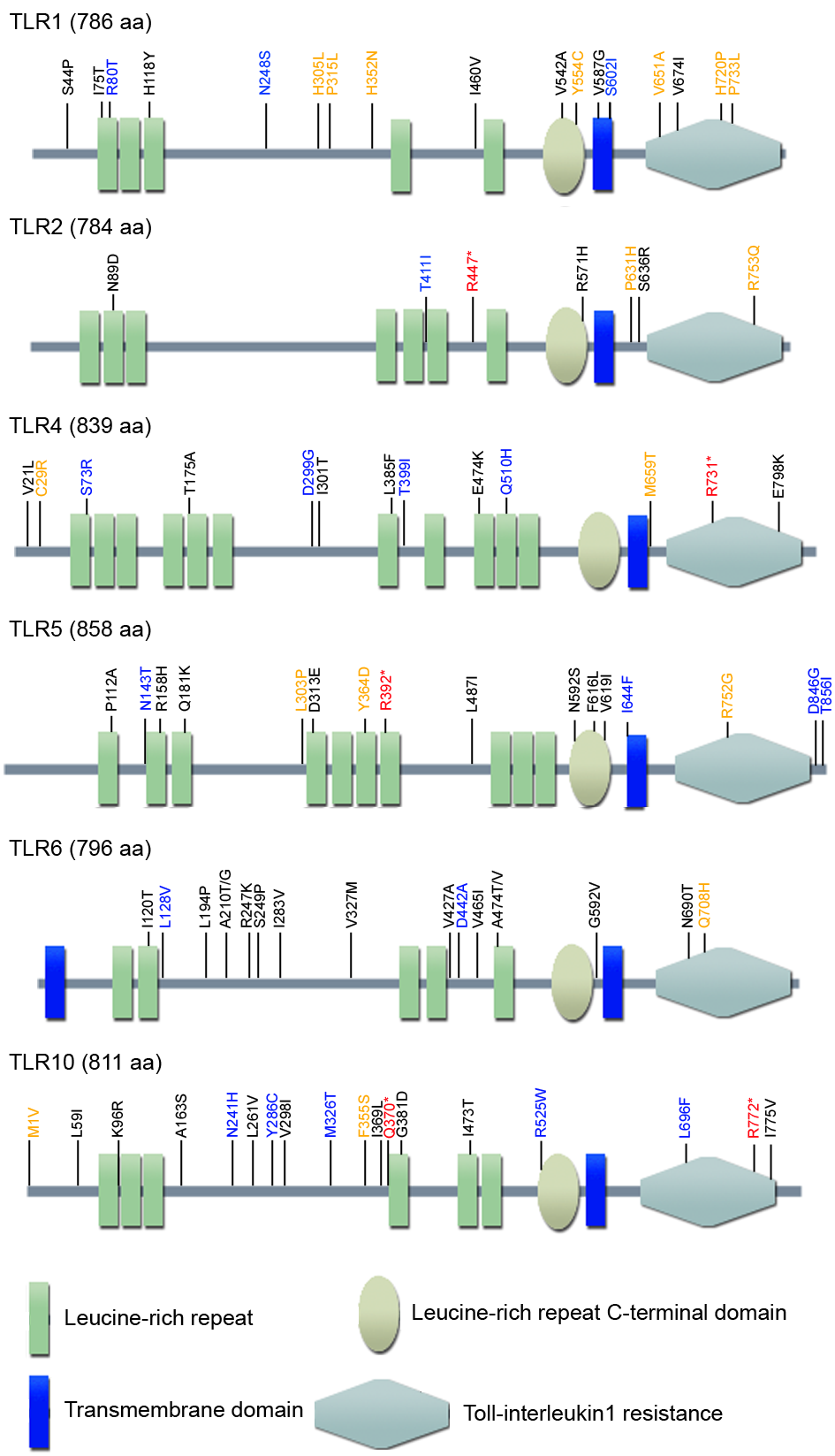

Supplement: Figure S10 — Protein domain architecture of the cell-surface expressed TLRs. Nonsynonymous mutations in black, blue and orange correspond to those considered as benign, possibly damaging and probably damaging. Variants in red correspond to stop mutations. The identification of the protein domains of the different TLR members was defined using the SMART program [103]. (8.20 MB DOC) [file pgen.1000562.s010.doc]
