# Supplementary material for: Evolutionary Dynamics of Human Toll-Like Receptors and Their Different Contributions to Host Defense
Source: PLoS Genet. 2009 Jul 17;5(7):e1000562. doi: 10.1371/journal.pgen.1000562 (PMC2702086; doi:10.1371/journal.pgen.1000562)
Supplement: Figure S5 — Inferred haplotypes for the TLR10-TLR1-TLR6 gene cluster. Haplotype composition and frequency distribution in (A) Africans, (B) Europeans, and (C) East-Asians. The chimpanzee sequence was used to deduce the ancestral state at each position. Yellow columns correspond to nonsynonymous mutations. The frequency of each haplotype in the different populations studied is presented in the right of the figure. Haplotypes identified as being under positive selection by the DIND test are presented in red. Only haplotypes appearing more than once in each of the populations are shown. (0.06 MB DOC) [file pgen.1000562.s005.doc]

**Figure S5**

**A**

**B**

**C**
