# Supplementary material for: Evolutionary Dynamics of Human Toll-Like Receptors and Their Different Contributions to Host Defense
Source: PLoS Genet. 2009 Jul 17;5(7):e1000562. doi: 10.1371/journal.pgen.1000562 (PMC2702086; doi:10.1371/journal.pgen.1000562)
Supplement: Table S1 — Details on sequenced regions and fragments for the 10 human TLRs. (0.28 MB DOC) [file pgen.1000562.s011.doc]

**Table S1. Details on sequenced regions and fragments for the 10 human TLRs**

| **Gene** | **Chromosomal location^a^** | **Sequenced fragments^b^** | **Sequenced lenght^c^** | **Exonic^c^** | **Non-exonic^c^** |
| --- | --- | --- | --- | --- | --- |
| TLR1 (uc003gtl.1) | chr4:38,474,271-38,482,807 | -6,820 : -5,402 | 5,355 | 2,849 | 2,506 |
|  |  | -2,802 : -1,700 |  |  |  |
|  |  | -214 : 2,621 |  |  |  |
| TLR2 (uc003ins.1) | chr4:154,828,504-154,846,692 | -15,907 : -14,760 | 3,804 | 2,605 | 1,199 |
|  |  | 1 : 2,657 |  |  |  |
| TLR3 (uc003iyq.1) | chr4:187,227,303-187,243,246 | -8,320 : -7,324 | 5,842 | 3,039 | 2,803 |
|  |  | -70 : 1,160 |  |  |  |
|  |  | 1,319 : 2,530 |  |  |  |
|  |  | 5,700 : 7,617 |  |  |  |
|  |  | 8,025 : 8,513 |  |  |  |
| TLR4 (uc004bjz.1) | chr9:119,506,431-119,519,587 | -1,143 : 191 | 7,464 | 3,814 | 3,650 |
|  |  | 3,545 : 4,932 |  |  |  |
|  |  | 7,799 : 12,542 |  |  |  |
| TLR5 (uc001hnv.1) | chr1:221,350,207-221,383,247 | -24,359 : -23,939 | 6,794 | 3,104 | 3,690 |
|  |  | -23,387 : -21,772 |  |  |  |
|  |  | -21,581 : -19,539 |  |  |  |
|  |  | -54 : 2,663 |  |  |  |
| TLR6 (uc003gtm.1) | chr4:38,504,803-38,507,555 | -1,093 : 3,300 | 4,393 | 2,753 | 1,640 |
| TLR7 (uc004cvc.1) | chrX:12,795,123-12,818,401 | -1,441 : 507 | 5,541 | 3,285 | 2,256 |
|  |  | 17,487 : 21,080 |  |  |  |
| TLR8 (uc004cvd.1) | chrX:12,834,679-12,851,209 | -4,940 : -3,517 | 6,034 | 3,468 | 2,566 |
|  |  | -753 : 333 |  |  |  |
|  |  | 8,597 : 12,122 |  |  |  |
| TLR9 (uc003dda.1) | chr3:52,230,138-52,235,219 | -1,556 : 4,710 | 6,266 | 3,868 | 2,398 |
| TLR10 (uc003gtj.1) | chr4:38,450,647-38,460,984 | -8,432 : -7,323 | 4,813 | 3,484 | 1,329 |
|  |  | -755 : 2,949 |  |  |  |

^a^ gh18 coordinates

^b^ Positions are relative to the start coding site of the corresponding isoform

^c^ Length in base pairs
