# Supplementary material for: Evolutionary Dynamics of Human Toll-Like Receptors and Their Different Contributions to Host Defense
Source: PLoS Genet. 2009 Jul 17;5(7):e1000562. doi: 10.1371/journal.pgen.1000562 (PMC2702086; doi:10.1371/journal.pgen.1000562)
Supplement: Table S2 — Genomic features and mean diversity indices of the 20 independent noncoding genomic regions. (0.10 MB DOC) [file pgen.1000562.s012.doc]

**Table S2. Genomic features and mean diversity indices of the 20 independent noncoding genomic regions**

|  |  |  | **Africa (N=126) ^a^** | | | | |  | **Europe (N=94) ^a^** | | | | |  | **East-Asia (N=96) ^a^** | | | | |
| --- | --- | --- | --- | --- | --- | --- | --- | --- | --- | --- | --- | --- | --- | --- | --- | --- | --- | --- | --- |
|  | **Position** | **lengh (bp)** | **S^b^** | **π^c^** | ***TD*^d^** | ***F*^e^** | ***H*^f^** |  | **S^b^** | **π^c^** | ***TD*^d^** | ***F*^e^** | ***H*^f^** |  | **S^b^** | **π^c^** | ***TD*^d^** | ***F*^e^** | ***H*^f^** |
| PR04 | Chr1: 106787624-106788951 | 1328 | 5 | 6.23 | -0.21 | -0.14 | 0.62 |  | 3 | 2.13 | -0.94 | -1.93 | 0.24 |  | 5 | 3.71 | -1.06 | -2.10 | 0.35 |
| PR05 | Chr2: 76163330-76164600 | 1271 | 13 | 16.15 | -0.38 | 0.50 | 1.13 |  | 9 | 26.06 | 2.22 | 1.93 | 0.78 |  | 9 | 12.37 | -0.26 | 0.92 | -1.68 |
| PR06 | Chr2: 117090864-117092163 | 1300 | 11 | 12.04 | -0.58 | -1.80 | -2.02 |  | 7 | 15.70 | 1.16 | 0.72 | -0.46 |  | 8 | 13.77 | 0.36 | -0.12 | -0.32 |
| PR07 | Chr3: 20665087-20666459 | 1373 | 8 | 5.14 | -1.23 | -2.78 | 0.56 |  | 6 | 8.92 | 0.10 | -0.57 | 0.79 |  | 6 | 8.09 | -0.11 | -0.66 | -1.51 |
| PR08 | Chr3: 146671295-146672665 | 1371 | 4 | 5.58 | 0.07 | -0.22 | 0.60 |  | 8 | 7.47 | -0.84 | -1.85 | 0.63 |  | 4 | 7.85 | 0.76 | 1.04 | 0.70 |
| PR09 | Chr4: 29547438-29548693 | 1256 | 9 | 11.20 | -0.37 | -0.95 | -0.26 |  | 6 | 8.97 | -0.09 | -0.65 | -1.10 |  | 5 | 7.80 | 0.01 | 0.83 | -0.98 |
| PR10 | Chr4: 179693433-179694758 | 1326 | 9 | 13.91 | 0.26 | -0.68 | -0.37 |  | 9 | 13.74 | 0.09 | 0.49 | -0.53 |  | 8 | 8.54 | -0.67 | 0.09 | -1.84 |
| PR14 | Chr6: 91757485-91758784 | 1300 | 4 | 3.63 | -0.69 | -2.48 | 0.29 |  | 3 | 2.84 | -0.67 | -1.84 | -0.92 |  | 1 | 2.75 | 0.96 | 0.74 | -0.84 |
| PR16 | Chr7: 13052349-13053749 | 1401 | 13 | 15.54 | -0.25 | 0.09 | 0.50 |  | 10 | 13.50 | -0.08 | 1.03 | -0.11 |  | 12 | 19.48 | 0.45 | 0.36 | 1.06 |
| PR17 | Chr7: 118479068-118480417 | 1350 | 11 | 5.25 | -1.65 | -2.26 | -1.22 |  | 6 | 5.40 | -0.86 | -0.95 | 0.41 |  | 2 | 0.90 | -1.07 | -1.24 | 0.02 |
| PR18 | Chr8: 5139032-5140287 | 1256 | 24 | 27.07 | -0.67 | -2.29 | -0.59 |  | 12 | 13.84 | -0.69 | -0.59 | -1.23 |  | 7 | 14.98 | 0.90 | 1.30 | -1.04 |
| PR20 | Chr8: 137174739-137176020 | 1282 | 10 | 13.44 | -0.17 | 0.41 | -1.10 |  | 6 | 11.34 | 0.54 | -0.40 | 0.69 |  | 4 | 12.49 | 2.11 | 1.55 | 0.59 |
| PR21 | Chr9: 11567765-11569154 | 1390 | 10 | 9.45 | -0.72 | 0.18 | -1.09 |  | 7 | 6.19 | -0.88 | -1.45 | -2.50 |  | 7 | 3.26 | -1.57 | -3.10 | 0.38 |
| PR25 | Chr11: 80750645-80751984 | 1340 | 9 | 8.36 | -0.79 | 0.08 | -0.27 |  | 8 | 5.42 | -1.31 | -0.17 | -0.96 |  | 4 | 5.28 | -0.18 | 0.68 | -1.11 |
| PR26 | Chr11: 96959343-96960672 | 1330 | 7 | 2.50 | -1.68 | -2.58 | 0.31 |  | 5 | 3.41 | -1.15 | 0.38 | 0.39 |  | 5 | 2.67 | -1.36 | -1.38 | 0.33 |
| PR30 | Chr14: 82997591-82998935 | 1345 | 9 | 10.14 | -0.44 | -0.98 | -1.13 |  | 7 | 9.09 | -0.25 | -1.20 | 0.23 |  | 6 | 8.73 | 0.01 | -0.61 | 0.20 |
| PR32 | Chr15: 84958820-84960120 | 1301 | 8 | 8.22 | -0.65 | 0.05 | 0.49 |  | 4 | 11.85 | 1.95 | 1.49 | 0.46 |  | 5 | 9.94 | 0.70 | 0.26 | 0.44 |
| PR33 | Chr16: 58084866-58086215 | 1350 | 14 | 23.35 | 0.58 | 0.08 | 2.24 |  | 10 | 25.88 | 2.03 | 1.90 | -0.11 |  | 9 | 23.15 | 1.97 | 1.83 | -0.76 |
| PR36 | Chr18: 26002122-26003321 | 1200 | 9 | 16.32 | 0.43 | -0.02 | 0.83 |  | 5 | 14.75 | 1.75 | 1.50 | 0.70 |  | 5 | 13.32 | 1.38 | 0.52 | -0.15 |
| PR42 | Chr20: 53069630-53070976 | 1347 | 11 | 10.84 | -0.71 | -0.28 | 1.10 |  | 3 | 4.44 | 0.04 | 0.68 | -0.82 |  | 3 | 1.21 | -1.30 | 0.19 | -1.74 |
| **Mean** |  | **1321** | **9.9** | **11.22** | **-0.49** | **-0.80** | **0.03** |  | **6.7** | **10.55** | **0.10** | **-0.07** | **-0.17** |  | **5.75** | **9.01** | **0.10** | **0.06** | **-0.39** |

^a^Number of chromosomes analyzed. For the X-linked *TLR7* and *TLR8*, the figures are 88, 68 and 74 for Africans, Europeans and East-Asians, respectively. ^b^Number of segregating sites (excluding indels). ^c^Number of haplotypes. ^d^Number of amino acid-altering mutations. ^e^Nucleotide diversity (x10^-4^). ^f^Haplotype diversity.
