# Supplementary material for: Evolutionary Dynamics of Human Toll-Like Receptors and Their Different Contributions to Host Defense
Source: PLoS Genet. 2009 Jul 17;5(7):e1000562. doi: 10.1371/journal.pgen.1000562 (PMC2702086; doi:10.1371/journal.pgen.1000562)
Supplement: Table S5 — Convergence and summary statistics of the marginal posterior distribution of ω estimations across 10 MCMC Chains with overdispersed starting points. (0.04 MB DOC) [file pgen.1000562.s015.doc]

**Table S5**. Convergence and summary statistics of the marginal posterior distribution of ω estimations across 10 MCMC Chains with overdispersed starting points.

|  | **Convergence States** | |  | **Posterior Distribution** | |  | **Quantiles** | |
| --- | --- | --- | --- | --- | --- | --- | --- | --- |
| **Gene^a^** | **rejection rate** | **Geban statistic^b^** |  | **mean** | **SD^c^** |  | **2.50%** | **97.50%** |
| TLR1 | 0.54 | 1.0000 |  | 1.11 | 0.31 |  | 0.59 | 2.00 |
| TLR2 | 0.50 | 1.0000 |  | 0.55 | 0.46 |  | 0.21 | 1.29 |
| **TLR3** | 0.51 | 0.9999 |  | 0.39 | 0.38 |  | 0.18 | 0.80 |
| TLR4 | 0.52 | 1.0001 |  | 0.88 | 0.35 |  | 0.43 | 1.70 |
| TLR5 | 0.54 | 1.0000 |  | 0.69 | 0.31 |  | 0.36 | 1.22 |
| TLR6 | 0.54 | 0.9999 |  | 0.78 | 0.30 |  | 0.42 | 1.38 |
| **TLR7** | 0.48 | 1.0000 |  | 0.32 | 0.50 |  | 0.12 | 0.81 |
| **TLR8** | 0.48 | 0.9998 |  | 0.09 | 0.63 |  | 0.02 | 0.27 |
| **TLR9** | 0.48 | 0.9999 |  | 0.21 | 0.49 |  | 0.07 | 0.53 |
| TLR10 | 0.54 | 1.0001 |  | 0.83 | 0.31 |  | 0.43 | 1.48 |

^a^ Genes in bold correspond to those which posterior distributions is significantly lower than 1.

^b^ If the chain have converged, values should be close to 1.

^c^ Posterior standard deviations.
