# Supplementary material for: Evolutionary Dynamics of Human Toll-Like Receptors and Their Different Contributions to Host Defense
Source: PLoS Genet. 2009 Jul 17;5(7):e1000562. doi: 10.1371/journal.pgen.1000562 (PMC2702086; doi:10.1371/journal.pgen.1000562)
Supplement: Table S6 — Convergence and summary statistics of the marginal posterior distribution of γ estimations across 10 MCMC Chains with overdispersed starting points. (0.04 MB DOC) [file pgen.1000562.s016.doc]

**Table S6**. Convergence and summary statistics of the marginal posterior distribution of γ estimations across 10 MCMC Chains with overdispersed starting points.

|  | **Convergence States** | |  | **Posterior Distribution** | |  | **Quantiles** | |
| --- | --- | --- | --- | --- | --- | --- | --- | --- |
| **Gene^a^** | **rejection rate** | **Geban statistic^b^** |  | **mean** | **SD^c^** |  | **2.50%** | **97.50%** |
| **TLR1** | 0.54 | 1.0005 |  | -0.80 | 0.39 |  | -1.54 | -0.02 |
| TLR2 | 0.50 | 1.0002 |  | -0.62 | 0.54 |  | -1.65 | 0.47 |
| TLR3 | 0.51 | 1.0000 |  | -0.10 | 0.49 |  | -0.97 | 0.96 |
| **TLR4** | 0.52 | 0.9999 |  | -1.10 | 0.49 |  | -2.11 | -0.18 |
| TLR5 | 0.54 | 0.9999 |  | -0.52 | 0.39 |  | -1.23 | 0.30 |
| TLR6 | 0.54 | 0.9997 |  | -0.45 | 0.37 |  | -1.15 | 0.33 |
| TLR7 | 0.48 | 1.0001 |  | -0.06 | 0.61 |  | -1.09 | 1.31 |
| TLR8 | 0.48 | 0.9999 |  | 0.14 | 0.74 |  | -1.10 | 1.82 |
| TLR9 | 0.48 | 0.9997 |  | 0.49 | 0.70 |  | -0.63 | 2.14 |
| **TLR10** | 0.54 | 1.0000 |  | -1.13 | 0.42 |  | -2.00 | -0.32 |

^a^ Genes in bold correspond to those which posterior distributions is significantly lower than 0.

^b^ If the chain have converged, values should be close to 1.

^c^ Posterior standard deviations.
