# Supplementary material for: Evolutionary Dynamics of Human Toll-Like Receptors and Their Different Contributions to Host Defense
Source: PLoS Genet. 2009 Jul 17;5(7):e1000562. doi: 10.1371/journal.pgen.1000562 (PMC2702086; doi:10.1371/journal.pgen.1000562)
Supplement: Table S7 — Prediction of the fitness effect for nonsynonymous variants identified among the different TLRs. (0.14 MB DOC) [file pgen.1000562.s017.doc]

**Table S7**. Prediction of the fitness effect for non-synonymous variants identified among the different TLRs.

| **Gene** | **ATG position** | **rs number** | **amino-acid Change** | **Polyphen** |
| --- | --- | --- | --- | --- |
| TLR1 | 130 |  | S44P | benign |
| TLR1 | 224 |  | I75T | benign |
| TLR1 | 239 | rs5743611 | R80T | possibly damaging |
| TLR1 | 352 | rs5743612 | H118Y | benign |
| TLR1 | 743 | rs4833095 | N248S | possibly damaging |
| TLR1 | 914 | rs3923647 | H305L | probably damaging |
| TLR1 | 944 | rs5743613 | P315L | probably damaging |
| TLR1 | 1054 |  | H352N | probably damaging |
| TLR1 | 1378 |  | I460V | benign |
| TLR1 | 1625 |  | V542A | benign |
| TLR1 | 1661 |  | Y554C | probably damaging |
| TLR1 | 1760 | rs5743617 | V587G | benign |
| TLR1 | 1805 | rs5743618 | S602I | possibly damaging |
| TLR1 | 1952 |  | V651A | probably damaging |
| TLR1 | 2020 |  | V674I | benign |
| TLR1 | 2159 |  | H720P | probably damaging |
| TLR1 | 2198 | rs5743621 | P733L | probably damaging |
| TLR2 | 265 |  | N89D | benign |
| TLR2 | 1232 | rs5743699 | T411I | possibly damaging |
| TLR2 | 1339 |  | R447stop | nonsense |
| TLR2 | 1712 |  | R571H | benign |
| TLR2 | 1892 | rs5743704 | P631H | probably damaging |
| TLR2 | 1906 |  | S636R | benign |
| TLR2 | 2258 | rs5743708 | R753Q | probably damaging |
| TLR3 | 346 |  | D116N | possibly damaging |
| TLR3 | 5863 |  | T266A | benign |
| TLR3 | 5905 |  | D280N | benign |
| TLR3 | 5986 | rs5743317 | Y307D | benign |
| TLR3 | 6301 | rs3775291 | L412F | possibly damaging |
| TLR3 | 6994 |  | R643S | benign |
| TLR3 | 7276 | rs5743318 | S737T | benign |
| TLR3 | 8139 |  | R867Q | possibly damaging |
| TLR4 | 61 |  | V21L | benign |
| TLR4 | 85 |  | C29R | probably damaging |
| TLR4 | 4216 |  | S73R | possibly damaging |
| TLR4 | 8179 | rs16906079 | T175A | benign |
| TLR4 | 8552 | rs4986790 | D299G | possibly damaging |
| TLR4 | 8558 |  | I301T | benign |
| TLR4 | 8811 | rs11536884 | L385F | benign |
| TLR4 | 8852 | rs4986791 | T399I | possibly damaging |
| TLR4 | 9076 | rs5030718 | E474K | benign |
| TLR4 | 9186 | rs5030719 | Q510H | possibly damaging |
| TLR4 | 9632 |  | M659T | probably damaging |
| TLR4 | 9847 |  | R731stop | nonsense |
| TLR4 | 10048 |  | E798K | benign |
| TLR5 | 334 | rs5744166 | P112A | benign |
| TLR5 | 428 | rs5744167 | N143T | possibly damaging |
| TLR5 | 473 |  | R158H | benign |
| TLR5 | 541 |  | Q181K | benign |
| TLR5 | 908 |  | L303P | probably damaging |
| TLR5 | 939 |  | D313E | benign |
| TLR5 | 1090 |  | Y364D | probably damaging |
| TLR5 | 1174 | rs5744168 | R392stop | nonsense |
| TLR5 | 1459 | rs5744171 | L487I | benign |
| TLR5 | 1775 | rs2072493 | N592S | benign |
| TLR5 | 1846 | rs5744174 | F616L | benign |
| TLR5 | 1855 |  | V619I | benign |
| TLR5 | 1930 | rs5744175 | I644F | possibly damaging |
| TLR5 | 2254 |  | R752G | probably damaging |
| TLR5 | 2537 | rs5744177 | D846G | possibly damaging |
| TLR5 | 2567 |  | T856I | possibly damaging |
| TLR6 | 359 | rs5743808 | I120T | benign |
| TLR6 | 382 |  | L128V | possibly damaging |
| TLR6 | 581 | rs5743809 | L194P | benign |
| TLR6 | 628 |  | A210T | benign |
| TLR6 | 629 |  | A210G | benign |
| TLR6 | 740 |  | R247K | benign |
| TLR6 | 745 | rs5743810 | S249P | benign |
| TLR6 | 847 |  | I283V | benign |
| TLR6 | 979 | rs3796508 | V327M | benign |
| TLR6 | 1280 | rs5743815 | V427A | benign |
| TLR6 | 1325 |  | D442A | possibly damaging |
| TLR6 | 1393 | rs5743816 | V465I | benign |
| TLR6 | 1420 | rs5743817 | A474T | benign |
| TLR6 | 1421 |  | A474V | benign |
| TLR6 | 1775 | rs5743617 | G592V | benign |
| TLR6 | 2069 |  | N690T | benign |
| TLR6 | 2124 |  | Q708H | probably damaging |
| TLR7 | 17962 | rs179008 | Q11L | benign |
| TLR7 | 19273 | rs5743781 | A448V | benign |
| TLR7 | 20753 |  | E941D | benign |
| TLR7 | 20893 |  | L988S | probably damaging |
| TLR8 | 8683 | rs5744077 | M28V | benign |
| TLR9 | 1227 |  | R5C | benign |
| TLR9 | 3802 | rs5743845 | R863Q | benign |
| TLR10 | 1 |  | M1V | probably damaging |
| TLR10 | 175 |  | L59I | benign |
| TLR10 | 287 |  | K96R | benign |
| TLR10 | 487 | rs11466649 | A163S | benign |
| TLR10 | 721 | rs11096957 | N241H | possibly damaging |
| TLR10 | 781 |  | L261V | benign |
| TLR10 | 857 |  | Y286C | possibly damaging |
| TLR10 | 892 | rs11466651 | V298I | benign |
| TLR10 | 977 | rs11466653 | M326T | possibly damaging |
| TLR10 | 1064 |  | F355S | probably damaging |
| TLR10 | 1105 | rs11096955 | I369L | benign |
| TLR10 | 1108 |  | Q370stop | nonsense |
| TLR10 | 1142 | rs11466655 | G381D | benign |
| TLR10 | 1418 | rs11466657 | I473T | benign |
| TLR10 | 1573 | rs11466658 | R525W | possibly damaging |
| TLR10 | 2088 |  | L696F | possibly damaging |
| TLR10 | 2314 |  | R772stop | nonsense |
| TLR10 | 2323 | rs4129009 | I775V | benign |
