# Supplementary material for: Evolutionary Dynamics of Human Toll-Like Receptors and Their Different Contributions to Host Defense
Source: PLoS Genet. 2009 Jul 17;5(7):e1000562. doi: 10.1371/journal.pgen.1000562 (PMC2702086; doi:10.1371/journal.pgen.1000562)
Supplement: Table S8 — Expected mean values of sequenced-based neutrality tests considering the demographic model of Voight et al. (2005). (0.04 MB DOC) [file pgen.1000562.s018.doc]

**Table S8.** Expected mean values of sequenced-based neutrality tests considering the demographic model of Voight *et al.* (2005)

|  | **Africa (N=126) ^a^** | | |  | **Europe (N=94) ^a^** | | |  | **East-Asia (N=96) ^a^** | | |
| --- | --- | --- | --- | --- | --- | --- | --- | --- | --- | --- | --- |
|  | *TD*^b^ | *F*^c^ | *H*^d^ |  | *TD*^b^ | *F*^c^ | *H*^d^ |  | *TD*^b^ | *F*^c^ | *H*^d^ |
| 20 noncoding regions^e^ | **-0.49*** | **-0.8*** | 0.03 |  | 0.1 | -0.07 | -0.17 |  | 0.1 | 0.06 | **-0.39*** |
|  |  |  |  |  |  |  |  |  |  |  |  |
| Voight *et al*.’s model^f^ | -0.36 | -0.69 | 0.03 |  | 0.09 | -0.06 | -0.18 |  | 0.08 | -0.07 | -0.17 |
| (95% CI) | (0.03:-0.76) | (-0.15:-1.26) | (0.52:-0.67) |  | (0.54:-0.35) | (0.42:-0.56) | (0.28:-0.76) |  | (0.53:-0.37) | (0.37:-0.58) | (0.24:-0.76) |

^a^Number of chromosomes analyzed. ^b^Tajima’s *D*. ^c^Fu and Li’s *F**. ^d^Fay and Hu’s *H*. ^e^Observed mean values of sequenced-based neutrality tests across the 20 noncoding regions sequenced in our population panel. ^f^Mean values of sequenced-based neutrality tests using simulated data under the Voight *et al*.’s demographic model, which considers (i) a bottleneck in non-African populations starting 40,000 YBP in an ancestral population of 9,450 individuals, and (ii) an exponential expansion in African populations (see Material and Methods for details). The 95% confidence intervals were calculated by simulating 1,000 sets of 20 sequences each (equivalent to the number of noncoding regions sequenced in our population panel; see Material and Methods for details)

**P* <0.05.Values in bold refer to significant deviations from a constant size demographic model.
