# Supplementary material for: Evolutionary Dynamics of Human Toll-Like Receptors and Their Different Contributions to Host Defense
Source: PLoS Genet. 2009 Jul 17;5(7):e1000562. doi: 10.1371/journal.pgen.1000562 (PMC2702086; doi:10.1371/journal.pgen.1000562)
Supplement: Table S9 — List of high-F ST SNPs observed in the 10 human TLRs. (0.08 MB DOC) [file pgen.1000562.s019.doc]

**Table S9**. List of high-*F*_ST_ SNPs observed in the 10 human TLRs.

| **Gene** | **SNP** | ***F*_st_ Comparison** | **SNP type** | **HTZ** | ***F*_st_** | ***P*-value** |
| --- | --- | --- | --- | --- | --- | --- |
| *TLR1* | 1805 | Africans vs Europeans | Non-synonymous | 0.34 | 0.54 | 0.0089 |
| *TLR8* | 10608 | Africans vs Europeans | Synonymous | 0.42 | 0.59 | 0.0193 |
|  |  |  |  |  |  |  |
| *TLR1* | -6399 | Africans vs East-Asians | non-coding | 0.33 | 0.48 | 0.0276 |
| *TLR1* | -6375 | Africans vs East-Asians | non-coding | 0.33 | 0.48 | 0.0276 |
| *TLR1* | -5748 | Africans vs East-Asians | non-coding | 0.33 | 0.48 | 0.0276 |
| *TLR1* | -5644 | Africans vs East-Asians | non-coding | 0.33 | 0.48 | 0.0276 |
| *TLR1* | -5567 | Africans vs East-Asians | non-coding | 0.33 | 0.48 | 0.0276 |
| *TLR1* | -5531 | Africans vs East-Asians | non-coding | 0.33 | 0.48 | 0.0276 |
| *TLR1* | -5490 | Africans vs East-Asians | non-coding | 0.33 | 0.48 | 0.0276 |
| *TLR1* | -2611 | Africans vs East-Asians | non-coding | 0.33 | 0.48 | 0.0279 |
| *TLR1* | -2461 | Africans vs East-Asians | non-coding | 0.32 | 0.47 | 0.0286 |
| *TLR1* | -2192 | Africans vs East-Asians | non-coding | 0.32 | 0.47 | 0.0286 |
| *TLR1* | -6010 | Africans vs East-Asians | non-coding | 0.31 | 0.44 | 0.0329 |
| *TLR10* | -260 | Africans vs East-Asians | non-coding | 0.30 | 0.42 | 0.0352 |
| *TLR10* | 2723 | Africans vs East-Asians | 3'-UTR | 0.30 | 0.42 | 0.0352 |
| *TLR1* | -2076 | Africans vs East-Asians | non-coding | 0.30 | 0.42 | 0.0359 |
| *TLR10* | 2323 | Africans vs East-Asians | Non-synonymous | 0.28 | 0.38 | 0.0396 |
| *TLR4* | 11381 | Africans vs East-Asians | non-coding | 0.17 | 0.24 | 0.0444 |
|  |  |  |  |  |  |  |
| *TLR7* | 17962 | Europeans vs East-Asians | Non-synonymous | 0.18 | 0.22 | 0.0137 |
| *TLR8* | -4824 | Europeans vs East-Asians | non-coding | 0.50 | 0.54 | 0.0186 |
| *TLR8* | -3808 | Europeans vs East-Asians | non-coding | 0.50 | 0.54 | 0.0186 |
| *TLR8* | -3679 | Europeans vs East-Asians | non-coding/Non-synonymous | 0.50 | 0.54 | 0.0186 |
| *TLR1* | 1805 | Europeans vs East-Asians | Non-synonymous | 0.39 | 0.47 | 0.0234 |
| *TLR8* | -4284 | Europeans vs East-Asians | non-coding | 0.31 | 0.37 | 0.0246 |
| *TLR8* | -746 | Europeans vs East-Asians | non-coding | 0.50 | 0.49 | 0.0282 |
| *TLR6* | 745 | Europeans vs East-Asians | Non-synonymous | 0.25 | 0.28 | 0.0438 |
|  |  |  |  |  |  |  |
| *TLR1* | 1805 | Overall | Non-synonymous | 0.27 | 0.49 | 0.0025 |
| *TLR7* | 21017 | Overall | Synonymous | 0.20 | 0.30 | 0.0211 |
| *TLR2* | -15731 | Overall | non-coding | 0.32 | 0.45 | 0.0214 |
| *TLR1* | -2299 | Overall | non-coding | 0.08 | 0.15 | 0.0315 |
| *TLR6* | 745 | Overall | Non-synonymous | 0.17 | 0.25 | 0.0375 |
